# The Separate Roles of Vascular Plants and *Sphagnum* Mosses in Regulating the Net CO_2_ Exchange in a Boreal Peatland During Key Phenological Phases

**DOI:** 10.1111/gcb.70834

**Published:** 2026-04-03

**Authors:** Antonia Hartmann, Kyohsuke Hikino, Lukas Guth, Gillian Simpson, Järvi Järveoja, Mats B. Nilsson, Matthias Peichl

**Affiliations:** ^1^ Department of Forest Ecology and Management Swedish University of Agricultural Sciences Umeå Sweden; ^2^ Thünen‐Institute of Climate‐Smart Agriculture Braunschweig Germany

**Keywords:** boreal mire, carbon cycle, climate change, net ecosystem exchange of carbon dioxide, plant functional groups, plant phenology

## Abstract

Boreal peatlands provide an important carbon store, which is highly susceptible to future changes in the global climate. Predictions of climate feedbacks on the peatland carbon balance require an in‐depth understanding of how vegetation dynamics and environmental conditions jointly govern the production and decomposition of organic matter. However, detailed knowledge on the separate roles of plant functional groups (PFGs) in regulating peatland production and respiration fluxes in response to various abiotic factors at sub‐seasonal scales is currently lacking. In this study, we used high‐temporal resolution CO_2_ flux data from an automated chamber system established across experimental vegetation removal plots to separate the production and respiration fluxes of vascular plants and *Sphagnum* mosses over three growing seasons (2021–2023) in a boreal peatland. We found that gross primary production (GPP) of *Sphagnum* mosses exceeded that of vascular plants during green‐up (average ratio: 1.18) and senescence (1.11), whereas vascular plants were the main contributor during the peak season (0.88). Vascular plants dominated autotrophic respiration (RA; 78%–93%) in all phenophases and contributed 38%–40% to growing season ecosystem respiration. For both PFGs, plant phenology was the primary driver for variations in GPP during green‐up, whereas photosynthetic photon flux density was most important in regulating GPP during the peak season and senescence. Vascular plants reached greater maximum GPP throughout all phenophases, whereas *Sphagnum* mosses had a higher initial light use efficiency during green‐up and senescence. Moss RA exhibited greater daytime temperature sensitivity than vascular plants during the peak season and senescence, but not during nighttime. These findings highlight that climate change effects on vegetation phenology and composition may strongly alter the peatland carbon cycle. Thus, understanding the separate roles of vascular plants and *Sphagnum* mosses in regulating production and respiration fluxes in different environmental conditions is crucial to improve predictions of northern peatland carbon cycle‐climate feedbacks.

## Graphical abstract

In this study, we disentangle the roles of plant functional groups in regulating CO_2_ production and respiration fluxes across key phenophases using automated chamber measurements in a boreal peatland. *Sphagnum* mosses dominate gross primary production (GPP) during green‐up and senescence, while vascular plants prevail during the peak season. Phenology is the main driver of GPP during green‐up, whereas radiation becomes more important later in the growing season. Moss respiration shows greater daytime temperature sensitivity than vascular plants during peak season and senescence. These findings indicate that climate‐driven changes in vegetation phenology and composition may strongly alter peatland carbon cycling.

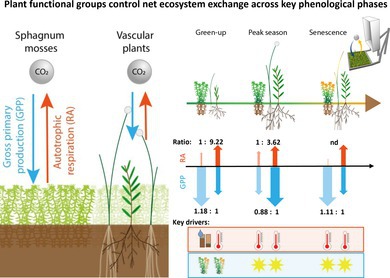

## 1 Introduction

Despite covering only around 3% of the earth's surface, peatlands store around 500–700 Gt of carbon (Yu 2011), which is more than half of all carbon (C) in the atmosphere (Grace 2004). Boreal peatlands constitute approximately 90% of global peatlands (Yu 2011) with average contemporary C accumulation rates ranging from 20 to 70 g C m^−2^ year^−1^ (Dinsmore et al. 2010; Koehler et al. 2011; M. Nilsson et al. 2008; Olefeldt et al. 2012; Roulet et al. 2007). The fate of this C store in a changing global climate is, however, highly uncertain (Antala et al. 2022; Calvin et al. 2023; Dise 2009). This is primarily due to the limited understanding of how various peatland C cycle processes will respond to altered environmental conditions. Thus, there is a need to better understand the interactions of climate with the underlying components of the peatland carbon balance.

The dominant component of the peatland C cycle is the net ecosystem exchange (NEE) of carbon dioxide (CO_2_), which represents the balance between the CO_2_ uptake via plant photosynthesis (i.e., gross primary production, GPP) and the loss of CO_2_ via ecosystem respiration (ER), with the latter including contributions from plant autotrophic (RA) and soil heterotrophic (RH) respiration. Apart from the sensitivity of NEE to abiotic variables such as temperature, water table level and solar radiation (Peichl et al. 2014; Yao et al. 2022), vegetation composition, physiology and photosynthetic activity strongly regulate GPP and RA (Järveoja et al. 2018, 2020; Peichl et al. 2018) as well as RH via root exudates and litter quality (Ward et al. 2015; Zeh et al. 2019). With climatic changes proceeding at the fastest rates in high latitudes, the predicted changes in temperature and precipitation (Christensen and Christensen 2007) will likely have considerable consequences for vegetation dynamics and subsequently for the C balance of northern peatlands (Antala et al. 2022; Page and Baird 2016). Hence, understanding the role of vegetation dynamics in regulating NEE is crucial for estimating future peatland C cycle‐climate feedbacks.

While most previous studies have focused on exploring how abiotic factors control the temporal dynamics of peatland NEE (e.g., Gažovič et al. 2013; Helfter et al. 2015; Strachan et al. 2016; Yao et al. 2022), the effects from seasonal vegetation development are less well understood. However, several studies have recently highlighted that plant phenology (i.e., the recurring physiological change of plants during the growing season (Lieth 1974)) plays an important role in regulating the peatland C cycle (Järveoja et al. 2018; Koebsch et al. 2020; Kross et al. 2016; Linkosalmi et al. 2016; Peichl et al. 2015). Specifically, phenology may act as an important mediator for temperature and radiation effects on ecosystem GPP (Koebsch et al. 2020) and cause diel to seasonal shifts in the contribution of RA and RH to ER (Järveoja et al. 2018, 2020). Peatland vegetation is, however, composed of different plant functional groups (PFGs), which each have distinct phenology trajectories in response to different controls (Peichl et al. 2018). Thus, there is a need to understand the individual effects from different PFGs and how these jointly regulate the peatland CO_2_ exchange.

The main PFGs in northern peatlands are *Sphagnum* mosses and vascular plant types including graminoids and ericoid dwarf‐shrubs (Ward et al. 2009), and their contribution to ecosystem GPP has been reported to vary over the growing season (Korrensalo et al. 2017). Specifically, while most of the seasonal CO_2_ dynamics are commonly explained by shrub and graminoid species because of their larger photosynthetic capacity and phenological variation (Bubier et al. 1998; Gavazov et al. 2018; Laine et al. 2022; Leppälä et al. 2008; Peichl et al. 2018), the contribution of *Sphagnum* mosses to ecosystem GPP may be significant in early spring and autumn (Korrensalo et al. 2017). Furthermore, autotrophic respiration of *Sphagnum* mosses has been found to be less responsive to seasonal change than vascular plants (Armstrong et al. 2015), while others reported a stronger seasonality with high respiration rates of *Sphagnum* mosses towards the end of summer (Korrensalo et al. 2016). However, most of this knowledge is based on laboratory experiments (Korrensalo et al. 2016, 2017) or manual chamber measurements conducted in weekly to monthly time intervals (Armstrong et al. 2015; Gavazov et al. 2018; Laine et al. 2022; Leppälä et al. 2008; Peichl et al. 2018). While such studies have provided valuable insights highlighting the separate roles of PFGs, the inherent coarse timescales in most field studies are insufficient to provide a detailed understanding of how PFGs regulate the peatland NEE and its response to environmental variations on daily to sub‐seasonal scales.

The relative importance of NEE component fluxes and their drivers associated with different PFGs likely vary across different phenological phases (i.e., green‐up, peak season and senescence) in response to changes in plant physiology, C allocation patterns and sensitivity to environmental conditions (Järveoja et al. 2018; Savage et al. 2013; Wang et al. 2014). For instance, the seasonal increase and decline in vascular plant biomass across the three key phenophases strongly determines the seasonal variation in plant productivity and autotrophic respiration (Korrensalo et al. 2017; Peichl et al. 2018; Järveoja et al. 2018). In comparison, abiotic factors such as water table level (Riutta et al. 2007), temperature and radiation are more important controls on the seasonal variations in *Sphagnum* moss productivity and respiration (Gunnarsson 2005; Loisel et al. 2012). Furthermore, vascular plants release root exudates promoting microbial respiration, whereas *Sphagnum* moss tends to inhibit decomposition via release of secondary metabolites (Mastný et al. 2021). However, detailed knowledge on the separate responses of PFG‐specific production and respiration fluxes to abiotic variables during key phenological phases is currently lacking. This is primarily due to the lack of high‐temporal resolution data for PFG‐specific fluxes that are needed to disentangle the influence of relatively fast changing environmental variables (e.g., radiation and temperature) from that of the slower changes in photosynthetic biomass. Specifically, empirical estimates of the temporal variations in the net primary production (NPP) of *Sphagnum* mosses are entirely missing, as standard measuring techniques (i.e., mesh harvest, brushwire method; (Rydin and Jeglum 2013)) commonly quantify moss biomass production only on a growing season to annual timescale. Thus, there is a need for a detailed process‐based understanding of how biotic and abiotic factors in concert regulate the peatland NPP alongside with other NEE production and respiration components at daily to seasonal scales. This information is also essential to improve peatland C cycle models in order to more accurately simulate the peatland NEE response to future climatic changes (Qiu et al. 2018; Wu et al. 2013, 2016).

In this study, we used high‐temporal resolution CO_2_ flux data from an automated chamber system established over experimental vegetation removal plots to explore the separate roles of vascular plants and *Sphagnum* mosses in regulating the NEE component fluxes in a boreal peatland over three growing seasons. The main objectives were to: (i) partition NEE into its individual production (GPP and NPP) and respiration (ER, RH, RA) fluxes; (ii) determine the relative importance of vascular plants and *Sphagnum* mosses in regulating the NEE component fluxes during distinct phenological phases; (iii) compare environmental responses of the production and respiration fluxes from vascular plants and *Sphagnum* mosses during different phenological phases.

## 2 Material and Methods

### 2.1 Site Description

The study was performed at Degerö Stormyr (64°18 N, 19°55E; altitude 265 m a.s.l.), which is an Integrated Carbon Observation System (ICOS) ecosystem station and part of the Kulbäcksliden Research Infrastructure located in northern Sweden (Västerbotten county) (Noumonvi et al. 2023). The climate in this region is classified as continental subarctic (Dfc) according to Köppen classification with a 30‐year mean (1991‐2020) annual precipitation of 645 mm and annual air temperature of +3°C (Noumonvi et al. 2023). The peatland is defined as an oligotrophic minerogenic mire consisting of an irregular mosaic of carpets and lawns with sparse occurrence of hummocks. The vegetation consists primarily of the vascular species *Eriophorum vaginatum* L., *Trichophorum cespitosum* L. Hartm., *Scheuchzeria palustris* L., *Carex pauciflora* Lightf., *Andromeda polifolia* L., *Vaccinium oxycoccos* L. and the *Sphagnum* species *S. balticum* Russ. C.Jens, *S. lindbergii* Schimp., *S. majus* Russ C.Jens and *S. papillosum* Lindb (Laine et al. 2012; Nilsson et al. 2008).

### 2.2 Experimental Study Design

A custom‐made automated chamber system based on the design by Goulden and Crill (1997) and Bubier et al. (2003) has been in operation since 2014 in close vicinity to an eddy covariance (EC) tower (Järveoja et al. 2018). The system includes 12 chambers which are distributed in four replicate groups, each including three treatments: natural, vascular plant removal and total vegetation removal plots. The vascular plant removal plots (hereafter called “moss plots”) were established in Spring 2020, however, the 2020 growing season data were not considered in this study since initial artefacts from the clipping (i.e., moss plot respiration > ER) were noted. The total vegetation removal plots (hereafter “RH plots”) were established by clipping all vascular plant and photosynthetically active moss biomass (i.e., the upper ~5 cm) in Autumn 2018. In addition, both moss and RH plots were trenched (30 cm deep) at the beginning of every growing season to prevent lateral in‐growth of roots. Throughout the growing season, new emerging shoots were clipped. It is important to note that trenching or vegetation‐removal techniques commonly cannot entirely separate autotrophic respiration (RA) from the portion of heterotrophic respiration (RH) from microbial communities associated with roots (Bond‐Lamberty et al. 2011). Consequently, our RH estimates primarily reflect the decomposition of old organic matter, while underestimating the contribution of microbial respiration derived from recent labile C sources (the latter being allocated to RA in this partitioning approach). However, lateral inflow of plant‐derived labile C in the soil solution from the surrounding vegetated areas may somewhat mitigate this shortcoming. To maintain a similar surface albedo and thereby minimize potential effects on soil temperature and evaporation rates due to the missing vegetation cover, air‐ and water‐permeable artificial grass mats were placed on the RH plots. Elevated boardwalks were constructed to access the chambers without disturbance. During this study, the system was in operation during the frost‐free period from May 28 to October 18 2021, May 15 to October 24 2022 and May 18 to October 7 2023.

### 2.3 Description of the Automated Chamber Measurement System

The configuration of the automated chamber system was previously described in detail by Järveoja et al. (2018). In brief, each chamber comprises a rectangular aluminium frame with a headspace (45 × 45 × 15 cm) attached to the backside of two movable arms. The frame includes a skirt extending 10 cm below the peat surface and a water‐filled groove for airtight chamber closure. A water pump, activated by a digital timer, regularly refilled the groove with water from a tank. The moving chamber was constructed from transparent Lexan polycarbonate with 84% light transmission. Reflective aluminium foil covered the chambers on RH plots for dark measurements. The opening and closing of the chambers was driven by double‐acting pneumatic cylinders (Clippard Instrument Laboratory Inc., Cincinnati, OH, USA) connected to a continuous air supply from a compressor (Model 86R‐4B‐BEEAA, GAST Manufacturing Inc., Benton Harbor, MI, USA). Air sampling tubes entered the chamber headspace through the side of the frame, ensuring a fixed position of the inlet at ~10 cm above the surface. T‐shaped plug‐in connectors were fitted on the intake and return ends of the sampling tube which were positioned opposite each other to facilitate constant mixing within the headspace throughout the measurement. The chambers were connected in a closed loop to a Picarro isotope analyzer (G2201‐i Isotopic Analyzer; Picarro Inc., CA, USA) housed inside a climate‐controlled instrument cabin (Nielsen et al. 2019). Sample air was circulated between the analyzer and chambers via polypropylene tubing (Synflex 1300, 6.4 mm i.d., 20 m one‐way length) at 5 L/min, propelled by an external diaphragm pump (Model N811KTE, KNF Neuberger GmbH, Freiburg, Germany). From the main sampling loop, a subsample was diverted into the analyzer at 30–35 mL/min, measuring CO_2_, CH_4_, and water vapor concentrations at 2 Hz, without being returned to the main sample loop. An in‐line water trap upstream of the analyzer opened in regular intervals during the flushing of the tubing to prevent liquid water entry to the analyzer cell.

To facilitate both light and dark measurements at the vegetated (i.e., natural and moss) plots, an additional shading umbrella consisting of an aluminium base frame (1.2 × 1.6 m) and a movable dome‐shape structure was installed around each transparent chamber in 2020 (see Figure S1). The movable umbrella structure consisted of several u‐shaped aluminium bars to which an opaque and foldable fabric was attached. The closing and opening of the umbrella was operated by an actuator (WM8DA/14 mm WM actuators, WireMatic TruTorq AB, Stockholm, Sweden), powered by the compressor and controlled by a data logger (Model CR1000, Campbell Scientific Inc., Logan, UT, USA).

The complete measuring cycle was 2 h (except in 2023 when it was 4 h due to a parallel experiment), with a constant sequence from group one to four, and within each group from the natural to the moss and last to the RH plots. More details on the various steps within each measurement cycle for the vegetated and RH plots are described in the Supporting Information Section S1.

### 2.4 Ancillary Environmental Measurements

Ancillary environmental measurements included air temperature (*T*_a_), measured continuously 10 cm above the mire surface using thermocouple wires (Type K, PFA insulated, 0.25 mm diameter; Omega Engineering Inc., Norwalk, CT, USA) and photosynthetic photon flux density (PPFD) inside the chambers at each vegetated plot using quantum sensors (Model SQ‐500‐SS, Apogee Instruments Inc., Logan, UT, USA) with measurements recorded at 1 Hz frequency and stored as 30 s averages. Soil temperature (*T*_s_) was measured at 2 and 10 cm depths beneath each chamber frame with thermistor probes (Model TO3R, TOJO Skogsteknik, Bygdeå, Sweden) at 1 min frequency and stored as 5 min averages. Next to each chamber plot a water level data logger (Model 3001 Levelogger 5 LTC, Solinst Canada Ltd., Georgetown, ON, Canada) was installed in a perforated plastic tube to monitor water table level (WTL) relative to the soil surface every 5 min. Additional meteorological data (*T*_a_, *T*_s_, PPFD, relative humidity, air pressure, ustar, precipitation, and vapour pressure deficit) were obtained from a weather station (Vaisala Weather Transmitter WXT520) and the ICOS tower (Nilsson et al. 2025), both located within 50 m from the automated chamber system. These data were used to complement meteorological conditions and to fill gaps in the automated chamber environmental data. For the latter purpose, specific regression relationships between the ambient data from the climate station and the respective chamber variables were developed to separately estimate *T*_a_ and PPFD inside (i.e., during chamber closure) and outside the chamber (i.e., ambient conditions).

### 2.5 Vegetation Inventory and Phenological Measurements

We used a digital image archive from a phenology camera (Canon A810 in 2021–2023 and Mobotix M26B‐6D in 2023, both with the same field of view) installed in close proximity to the chamber system area, to estimate a greenness index as a proxy for vegetation phenology at the ecosystem level. For this purpose, we defined a fixed area of interest in the images which spanned a mixture of both vascular plants and *Sphagnum* mosses. Average pixel values within this region of interest (i.e., digital numbers (0–255) of the red (R), green (G) and blue (B) image channels) were then used to calculate a greenness index (i.e., the green chromatic coordinate, gcc; Equation 1)

(1) $$\mathrm{gcc}=\frac{G}{R+G+B},$$

The distinct phenophases of the growing season (i.e., green‐up, peak season and senescence) were defined with the thresholding method of Gu et al. (2009). This approach uses a combination of local maxima and minima in the first derivative of the seasonal gcc trajectory, the baseline and the maxline to identify the onset and end of each phenophase. This results in three key phenophases which correspond to plant development. Green‐up represents the transitional phase from the onset of leaf emergence (i.e., start of the growing season) to the near maximum of leaf emergence, followed by the peak season (i.e., period with peak biomass, maximum greenness), after which greenness and related plant functions decline during senescence until the end of the growing season (i.e., onset of dormancy).

In addition, vegetation inventory was conducted biweekly in all vegetated plots to determine the seasonal changes in green and brown vascular plant biomass in accordance with ICOS protocols (Gielen et al. 2018). The brushwire method (Rydin and Jeglum 2013) was used to obtain an alternative estimate of growing season moss biomass production.

### 2.6 Data Processing, Flux Calculation and Quality Control

The data processing and quality control for the chamber data closely followed the protocols outlined by Järveoja et al. (2018). In brief, the fluxes were determined from the linear change in headspace gas concentration over a 3‐minute measurement period, adjusted for air density using the ideal gas law:

(2) $$\begin{array}{c} F=\frac{S*p*V}{R*T*A} \end{array}$$

where *F* represents the instantaneous CO_2_ flux in μmol m^−2^ s^−1^, *S* denotes the slope (ppm s^−1^) of the linear fit, *p* is the air pressure at a fixed value of 1013 kPa, *R* is the universal gas constant of 8.3143 J mol^−1^ K^−1^, *T* signifies the average headspace air temperature during the measurement (*K*), *A* represents the area within the chamber frame in (m^2^), and *V* denotes the volume of the chamber headspace (m^3^). The chamber‐specific volume was determined by measuring the distance from the top of the chamber to the moss/peat surface and accounting for temporal changes in the WTL as described in Järveoja et al. (2018).

To ensure the highest quality in slope estimation, the first three mean concentration records following chamber closure were excluded, implementing a 30‐s “dead band” to eliminate possible disturbance effects immediately after closure. Following this, a linear slope was calculated from 10 mean concentration data points spanning a window of 1 min and 40 s. This process was repeated for six consecutive windows, each stepwise shifted by one datapoint over the remaining data collected during the 3‐min chamber closure period. From these six different slopes, the one with the highest coefficient of determination (R^2^) was selected as the final value.

Following this, *T*_a_ and PPFD inside and outside the chamber were calculated for the same final slope window. The quality control procedure commenced with a screening of the concentration data to identify periods of system failure, such as power outages or non‐functioning umbrellas. Subsequently, poor‐quality flux data were removed using a combination of root mean square error (RMSE) and *R* ^2^ as goodness‐of‐fit measures. Specifically, all measurements with RMSE > 0.5 and *R* ^2^ < 0.95 (*p* < 0.001) were discarded, with these thresholds determined through visual examination of the data (Figure S2). In this study, all fluxes are reported following the atmospheric sign convention, where positive values indicate emission and negative values indicate uptake. Therefore, sporadic negative fluxes in ER, RH, and nighttime NEE (PPFD < 20 μmol m^−2^ s^−1^) were also eliminated. Furthermore, RH fluxes were excluded when the water table level (WTL) exceeded 5 cm above the surface of the mire, as the experimental plots were flooded during such conditions, resulting in unrealistically high RH fluxes.

To address potential overestimation of flux rates during calm nights with stable atmospheric conditions (Brændholt et al. 2017; Görres et al. 2016; Lai et al. 2012; Riederer et al. 2014; Schneider et al. 2009), an additional correction and filtering of the nighttime data was applied following the approach described by Järveoja et al. (2018). In brief, this method is based on determining the 20‐s mean CO_2_ concentrations directly before and after chamber closure to derive a relationship between the concentration change and respective flux estimates. The slope of the linear regression line was used to adjust nighttime fluxes that were moderately disturbed, while measurements exhibiting a concentration change beyond a predefined threshold (i.e., > 20 ppm) were eliminated. Additional information regarding the correction and filtering methods applied to nighttime fluxes can be found in the Figure S3.

### 2.7 Gap Filling

In total, of all potential 2‐ or 4‐hourly flux measurements between 23% and 47% of the natural plots, 21%–46% of the moss plots and 52%–78% RH plots were missing in each growing season due to periods of system failure and the described quality filtering (see Table S1). These missing data were gap‐filled using eXtreme Gradient Boosting implemented through the “xgboost” package in RStudio 4.1.2 to obtain continuous daily time series and growing season sums. Separate models for each chamber NEE and ER or RH fluxes were developed using gcc, *T*_a_, *T*_s_ at 2 and 10 cm depths, WTL, PPFD, relative humidity, vapour pressure deficit, and measurement year as predictors. The coefficient of determination (R^2^) of predicted to gap‐filled fluxes for holdout sets during 10‐fold cross validation ranged between 0.63 and 0.86 (see Table S1; Figure S4).

### 2.8 Partitioning the Net CO_2_ Exchange Into Production and Respiration Fluxes

Based on the independent measurements of fluxes under ambient light and dark conditions on the natural plots, combined with the RH flux estimates, the following NEE component fluxes (GPP, NPP, RA) were derived using the mass balance approach:

(3) $$\begin{array}{c} \mathrm{GPP}=\mathrm{NEE}-\mathrm{ER} \end{array}$$

(4) $$\begin{array}{c} \mathrm{NPP}=\mathrm{NEE}-\mathrm{RH} \end{array}$$

(5) $$\begin{array}{c} \mathrm{RA}=\mathrm{ER}-\mathrm{RH} \end{array}$$

*Sphagnum* moss specific component fluxes (GPP_M_, NPP_M_ and RA_M_) were calculated based on the independent measurements of fluxes on the moss plot (moss+peat “MP”) under light (NEE_MP_) and dark conditions (ER_MP_), as well as the RH estimates:

(6) $$\begin{array}{c} {\mathrm{GPP}}_{M}={\mathrm{NEE}}_{\mathrm{MP}}-{\mathrm{ER}}_{\mathrm{MP}} \end{array}$$

(7) $$\begin{array}{c} {\mathrm{NPP}}_{M}={\mathrm{NEE}}_{\mathrm{MP}}-\mathrm{RH} \end{array}$$

(8) $$\begin{array}{c} {\mathrm{RA}}_{M}={\mathrm{ER}}_{\mathrm{MP}}-\mathrm{RH} \end{array}$$

Vascular plant production (GPP_V_, NPP_V_) and respiration fluxes (RA_V_) were estimated by subtracting the moss component fluxes from the ecosystem‐level fluxes.

(9) $$\begin{array}{c} {\mathrm{GPP}}_{V}=\mathrm{GPP}-{\mathrm{GPP}}_{M} \end{array}$$

(10) $$\begin{array}{c} {\mathrm{NPP}}_{V}=\mathrm{NPP}-{\mathrm{NPP}}_{M} \end{array}$$

(11) $$\begin{array}{c} {\mathrm{RA}}_{V}=\mathrm{ER}-{\mathrm{ER}}_{M} \end{array}$$

### 2.9 Environmental Response Functions

The sensitivity of the respiration fluxes (RA_M_, RA_V_, RH) to changes in day‐ and nighttime air temperature for each phenological phase was estimated using the exponential model by Lloyd and Taylor (1994):

(12) $$\begin{array}{c} R=R_{10}\exp^{E_{0}\left(\frac{1}{56.02}-\frac{1}{T-227.13}\right)} \end{array}$$

where *R* is RA_M_, RA_V_, or RH, *T* is air temperature, *R*_10_ represents base respiration at 10°C, and *E*_0_ represents the activation energy parameter.

The light response of ecosystem and PFG‐specific GPP was estimated using the hyperbolic relationship between GPP (ecosystem, moss and vascular) and PPFD:

(13) $$\begin{array}{c} \mathrm{GPP}=-\frac{\alpha *\text{PPFD}*A_{\max}}{\alpha *\text{PPFD}+A_{\max}} \end{array}$$

where *α* is the initial slope of the light response curve and *A*_max_ the maximum assimilation rate at light saturation in μmol CO_2_ m^−2^ s^−1^.

### 2.10 Statistical Analysis

The relative importance of the key environmental variables and phenology (i.e., gcc) in regulating GPP and autotrophic respiration fluxes of vascular plants and *Sphagnum* mosses during the main phenophases was examined using stepwise generalized linear models (GLM) with a log‐link and gamma distribution. A constant (i.e., the respective minimum plus 1) was added to the negative values of GPP for the logarithmic transformation. Potential explanatory variables were gcc, *T*_a_, WTL and PAR and their two‐ and three‐way interactions. Soil temperature at 2 and 10 cm depth were excluded because of their strong correlation with each other and with *T*_a_ and gcc (pearson correlation coefficient, *r* > 0.8). All input variables were standardized and centred. The GLM models were based on non‐gapfilled daily mean fluxes and only included days with a minimum of 66% of the 2‐hourly means (4‐hourly means in 2023) from actual measurements, and were fitted using RStudio 4.1.2.

## 3 Results

### 3.1 Environmental Conditions

**FIGURE 1:**
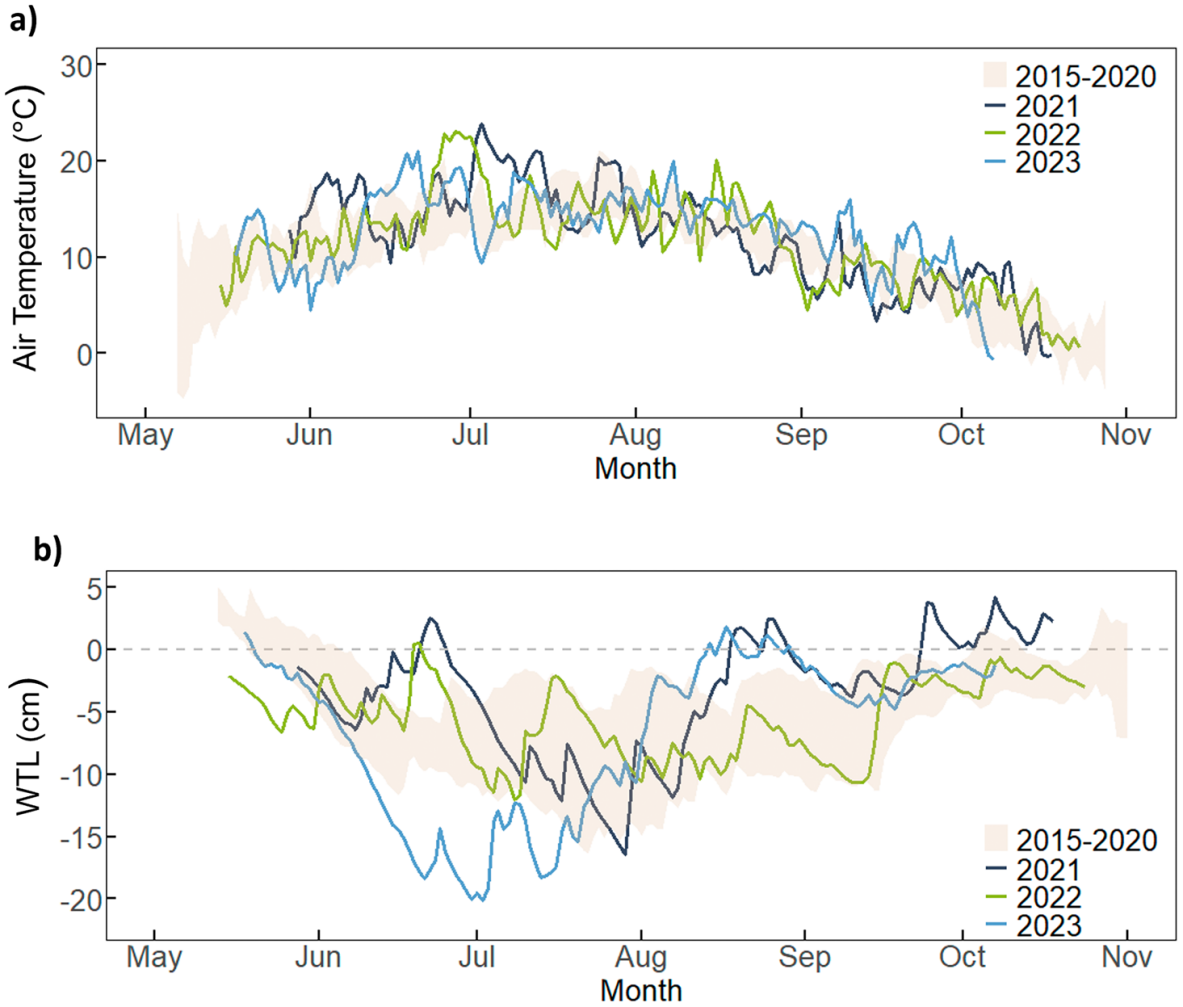
Daily means of (a) air temperature and (b) water table level (WTL) relative to the mire surface during the measurement periods in 2021–2023. Shaded band represents the 95% confidence interval of the long‐term means (2015–2020).

During the study period, the growing season mean air temperature was similar in 2021 and 2022 with 11.7°C and 11.3°C, whereas it was higher in 2023 with 13.0°C (Table 1). In 2021, green‐up was relatively cold with 10.4°C followed by a warm peak season (17.5°C) and cold senescence (9.5°C). During June 2023 the air temperature was 1.7°C warmer than the study site mean (2015–2020) (Figure 1a). This warm period was accompanied by a drought that caused an unusually low WTL that remained below the normal study site mean WTL range (i.e., ±1 standard error) from early June to late July, reaching a minimum of −20 cm at the end of June (Figure 1b). The growing season mean WTL of −6.8 cm in 2023 was therefore the lowest of the study period, compared to −3.8 and −5.3 cm in 2021 and 2022, respectively (Table 1).

**TABLE 1:** Ecosystem‐level start and end dates and length (days) of the main phenological phases (i.e., Green‐up, Peak, Senescence) presented alongside mean environmental conditions measured at the autochamber plots during the studied growing seasons of 2021–2023.

	Green‐up			Peak			Senescence			Growing season		
	2021	2022	2023	2021	2022	2023	2021	2022	2023	2021	2022	2023
DOY start	122	132	150	176	183	186	215	213	227	122	132	150
DOY end	176	183	186	215	213	227	285	275	268	285	275	268
Length	54	51	36	39	30	41	70	62	41	163	143	118
PPFD	344	398	406	383	324	293	153	173	208	271	251	293
*T*_a_	10.4	13.3	13.9	17.5	14.7	15.6	9.5	11.0	12.3	11.7	11.3	13.0
*T*_s_	10.9	11.4	11.9	16.3	15.2	14.3	10.7	11.8	12.8	12.1	11.5	12.4
WTL	−1.1	−4.3	−12.9	−8.7	−7.3	−9.7	−1.7	−6.8	−1.8	−2.5	−5.5	−6.8

Abbreviations: DOY, day of year; PPFD, photosynthetic photon flux density (μmol m^−2^ s^−1^); *T*_a_, air temperature (°C); *T*_s_, soil temperature at 10 cm depth (°C); WTL, water table level (cm).

### 3.2 Vegetation Phenology

**FIGURE 2:**
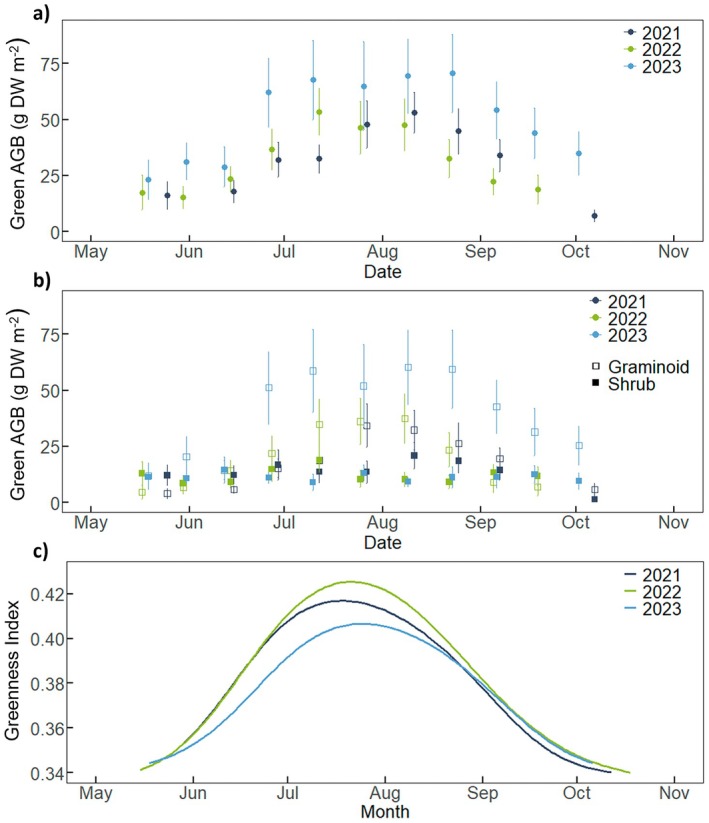
Seasonal development of (a) total vascular green aboveground biomass (AGB) (b) green AGB of graminoid species (unfilled rectangle) and dwarf shrub species (filled rectangle) and (c) Greenness Index (gcc) in 2021–2023. Error bars in (a) and (b) indicate ±1 standard error.

The length of growing season at the ecosystem‐level ranged from 118 to 163 days (Table 1). The aboveground vascular biomass pool reached its maximum during the peak season and was highest in 2023 with 70.6 g dry weight (DW) m^−2^ compared to 2021 (53.1 g DW m^−2^) and 2022 (53.5 g DW m^−2^) (Figure 2a; Table S2). This increase of the aboveground vascular biomass pool was due to a 1.4–1.7 times higher biomass pool of graminoid species compared to previous years (Figure 2b). In contrast, greenness index (i.e., gcc) during the peak growing season was lowest in 2023 and highest in 2022 (Figure 2c). Furthermore, gcc remained considerably lower during June to mid‐August of 2023 compared to the same months of the other years, coinciding with the early summer drought in 2023.

### 3.3 Seasonal Variations in Net CO_2_ Exchange and Its Underlying Component Fluxes

**FIGURE 3:**
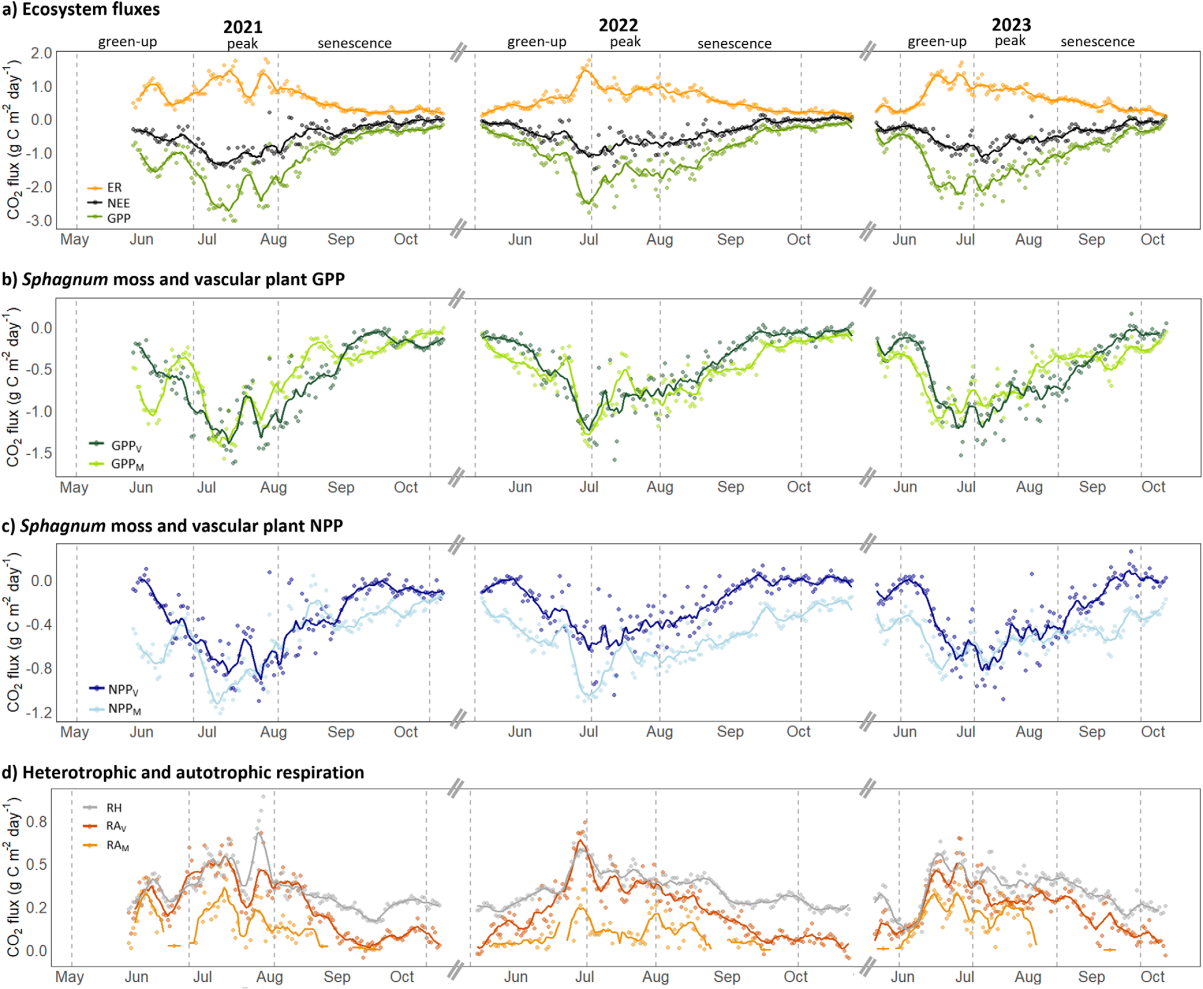
Daily sums of the (a) net ecosystem CO_2_ exchange (NEE) and its underlying component fluxes gross primary production (GPP) and ecosystem respiration (ER), (b) gross primary production of *Sphagnum* mosses (GPP_M_) and vascular plants (GPP_V_), (c) net primary production of mosses (NPP_M_) and vascular plants (NPP_V_) and (d) heterotrophic respiration (RH) and autotrophic respiration of mosses (RA_M_) and vascular plants (RA_V_) at the Degerö peatland during the growing seasons 2021–2023. Vertical dotted bars indicate the transitions (i.e., start or end dates) between the key phenological phases (green‐up, peak season and senescence).

The temporal patterns in daily ecosystem fluxes, NEE, GPP and ER differed between the measurement years (Figure 3a). Maximum net CO_2_ uptake was reached during the early peak season in all 3 years. In comparison, peak ER occurred already during green‐up in 2022 and 2023, with a steady decline in ER noted during the senescence. The seasonal pattern of daily GPP included two distinct productivity peaks (i.e., most negative values) during early peak season and during senescence, with an intermittent decrease during the peak season in all 3 years. Cumulative growing season sums of NEE, GPP and ER were similar in 2022 and 2023, whereas their sums for individual key phenophases differed by 1.5 to 2 times among the 3 years (Table 2). Specifically, peak season NEE, GPP and ER were higher in 2023 compared to 2022, while during senescence cumulative sums were lower in 2023. Cumulative sums for NEE and GPP during the peak season and senescence were highest in 2021.

**TABLE 2:** Cumulative sums (g C m^−2^) of the net ecosystem CO_2_ exchange (NEE) and its underlying component fluxes for ecosystem, *Sphagnum* mosses (_M_) and vascular plants (_V_) for the key phenological phases (i.e., Green‐up, Peak, Senescence) and growing season in 2021–2023, N.A. indicates that data were not available due to extended system failure.

	Green‐up			Peak			Senescence			Growing season		
	2021	2022	2023	2021	2022	2023	2021	2022	2023	2021	2022	2023
NEE	N.A.	−19.4	−20.3	−41.7	−24.3	−31.1	−20.7	−20.5	−11.2	N.A.	−64.2	−62.6
GPP	N.A.	−47.6	−52.5	−83.9	−51.6	−64.9	−49.7	−52.6	−28.5	N.A.	−151.9	−145.9
GPP_M_	N.A.	−26.0	−28.2	−40.1	−24.7	−28.8	−21.6	−31.3	−15.0	N.A.	−81.9	−72.0
GPP_V_	N.A.	−21.7	−24.2	−43.8	−26.9	−36.1	−28.1	−21.3	−13.5	N.A.	−69.9	−73.9
NPP	N.A.	−35.4	−33.3	−60.7	−37.9	−47.6	−40.5	−41.3	−24.1	N.A.	−114.6	−105.1
NPP_M_	N.A.	−25.3	−20.9	−33.7	−22.6	−24.0	−23.7	−31.5	−18.3	N.A.	−79.4	−63.2
NPP_V_	N.A.	−10.2	−12.4	−27.0	−15.2	−23.6	−16.8	−9.8	−5.8	N.A.	−35.2	−41.9
ER	N.A.	28.3	32.2	42.2	27.3	33.8	29.0	32.1	17.4	N.A.	87.7	83.4
RA	N.A.	12.2	19.1	23.2	13.8	17.3	9.2	11.3	4.4	N.A.	37.3	40.9
RA_M_	N.A.	0.7	7.3	6.4	2.1	4.8	−2.1	−0.3	−3.3	N.A.	2.5	8.8
RA_V_	N.A.	11.5	11.8	16.8	11.7	12.5	11.4	11.5	7.7	N.A.	34.8	32.0
RH	N.A.	16.1	13.0	19.0	13.5	16.5	19.8	20.8	13.0	N.A.	50.4	42.5

The temporal pattern in daily GPP_V_ and GPP_M_ differed between the key phenophases resulting in varying contribution to ecosystem GPP (Figure 3b). Specifically, while GPP_V_ commonly peaked around the transition of green‐up and peak phases and thereafter declined, GPP_M_ featured a double peak with highest productivity in late green‐up and senescence, and a pronounced intermittent decrease during the peak phase (Figure 3b). As a result, GPP_M_ was higher than GPP_V_ during most of the green‐up and senescence phases (GPP_M_:GPP_V_ ranging from 0.77 to 1.47), whereas GPP_V_ exceeded GPP_M_ during the peak phase (GPP_M_:GPP_V_ ranging from 0.80 to 0.97) in all 3 years. Averaged over the growing season, *Sphagnum* mosses contributed 49%–54% to the GPP in 2022 and 2023 (Table 2).

The temporal patterns in daily NPP_V_ and NPP_M_ overall reflected those of GPP_V_ and GPP_M_, respectively (Figure 3c). However, the reduction noted for GPP_M_ during the peak phase was less pronounced for NPP_M_. Furthermore, NPP_M_ exceeded NPP_V_ by up to 3 times during green‐up and senescence, with the difference being larger than that observed between GPP_M_ and GPP_V_ during these phenophases. As a result, the relative contribution of NPP_M_ to NPP varied from 50% to 60% during the peak phase to 59%–76% during green‐up and senescence across the 3 years. For the growing seasons of 2022 and 2023, the cumulative sums of NPP were −115 and −105 g C m^−2^, with *Sphagnum* mosses contributing 69% and 60% of NPP, respectively.

The partitioning of daily ER into its components RH, RA_V_, and RA_M_ revealed that RH was the highest ER component flux throughout most of the three growing seasons (Figure 3d). Among the RA components, RA_V_ was higher than RA_M_ throughout all growing seasons, except for occasional days during the peak seasons 2021 and 2023. Specifically, RA_V_ exceeded RA_M_ by 2–17 times during green‐up and up to 6 times during peak season across all 3 years. Averaged over the growing seasons, RA_M_ contributed 7%–22% to RA and 3%–11% to ER, while RA_V_ contributed 38%–40% to ER.

### 3.4 Environmental Controls of *Sphagnum* Moss and Vascular Plant Production and Respiration

**FIGURE 4:**
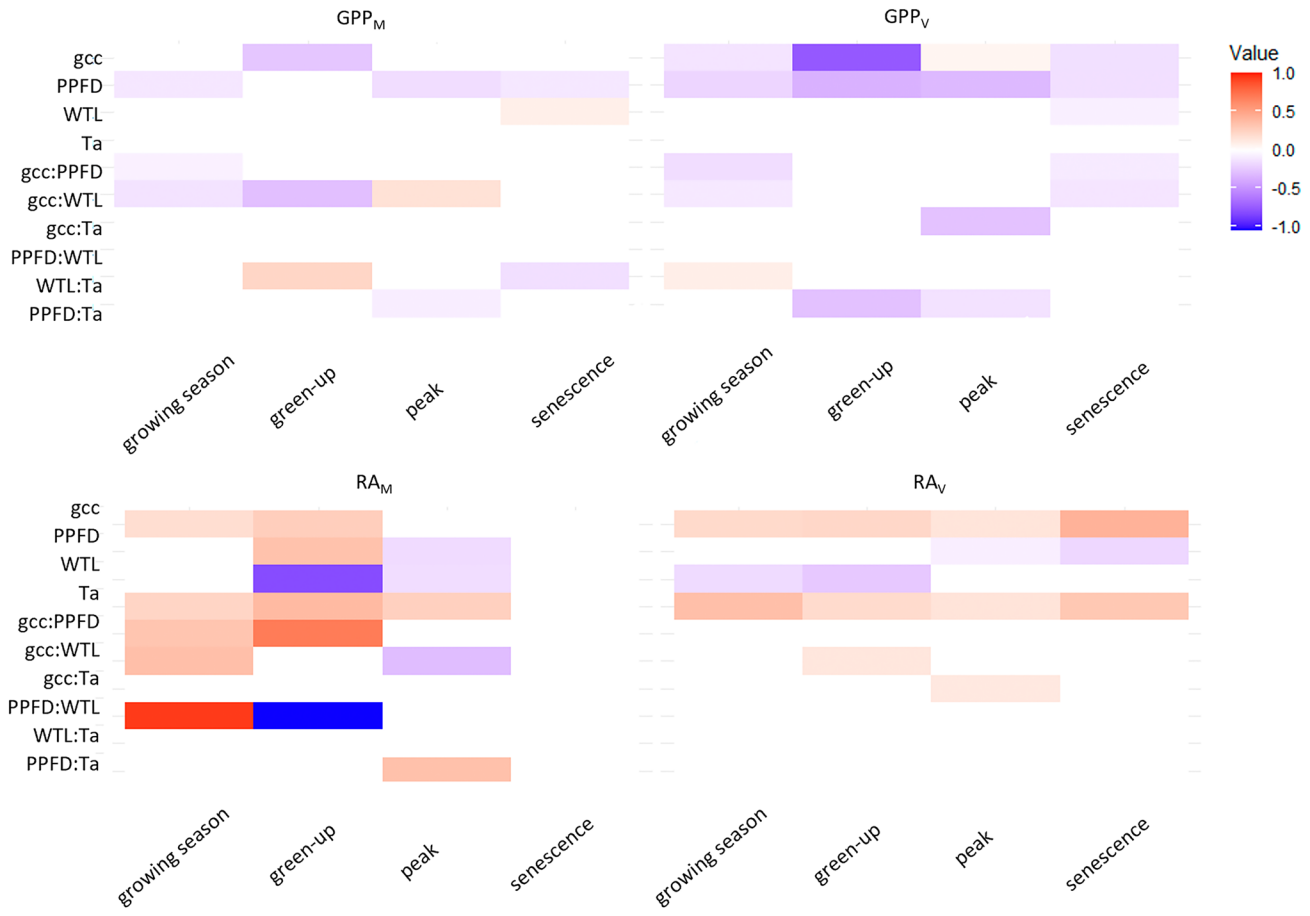
Heatmap illustrating the stepwise generalized mixed models results for abiotic (PPFD, photosynthetic photon flux density; *T*_a_, air temperature; WTL, water table level) and biotic factors (gcc, greenness index) explaining daily variation of gross primary production (GPP) and autotrophic respiration (RA) of *Sphagnum* mosses (_M_) and vascular plants (_V_). White cells indicate no effect size (*p* > 0.05) while red indicate a significant positive effect +1 and blue a significant negative effect. No significant model could be found for RA_M_ during senescence. Effect sizes of three‐way interaction can be found in Table S3.

The results of the GLM analysis suggested that gcc and PPFD were the dominant drivers of seasonal variations in daily *Sphagnum* moss and vascular plant GPP (Figure 4; Table S3). However, the relative importance of the environmental controls varied among the key phenophases. Specifically, for both *Sphagnum* moss and vascular plants, gcc explained most of the variation in the production fluxes during green‐up, while PPFD was the dominant control during the peak season and senescence. In contrast, the controlling factors for respiration fluxes showed less consistent patterns. Specifically, gcc, PPFD, *T*_a_, and WTL and their two‐ and three‐way interactions had varying effect sizes among phenophases for both RA_M_ and RA_V_. Furthermore, *T*_a_ was an important control of RA_M_ and RA_V_ in all phenophases. Water table level was the dominant factor for regulating RA_M_, especially during green‐up.

**FIGURE 5:**
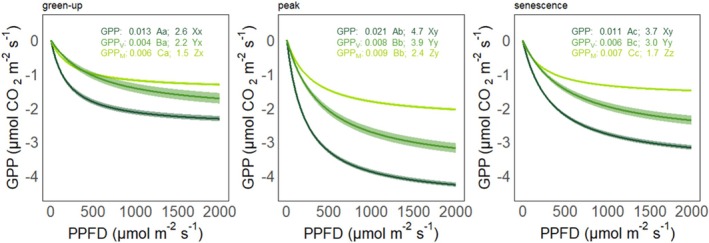
Light response curves for gross primary production of the ecosystem (GPP; dark green), vascular plants (GPP_V_; green) and *Sphagnum* mosses (GPP_M_; light green) during the key phenophases of green‐up, peak season and senescence averaged over 2021–2023. Lines show significant regression fits based on hyperbolic fit models (*p* < 0.05) with a band of 95% confidence interval. The values in the panels display the estimated parameters *α* and *A*_max_. Different capital and lowercase letters indicate significant differences between PFGs and among phenophases, respectively, based on the *z*‐test (*p* < 0.05).

The light response of GPP varied significantly between the PFGs and among the key phenophases (Figure 5). Specifically, the initial light use efficiency (i.e., *α*) was highest during the peak season and maximum photosynthetic capacity at saturated light conditions (i.e., *A*_max_) was lowest during green‐up for ecosystem GPP, GPP_V_ and GPP_M_. Vascular plants reached a greater maximum GPP (i.e., higher *A*_max_) compared to *Sphagnum* mosses throughout all phenophases, whereas under low light conditions, *Sphagnum* mosses had a higher initial light use efficiency during green‐up and senescence.

**FIGURE 6:**
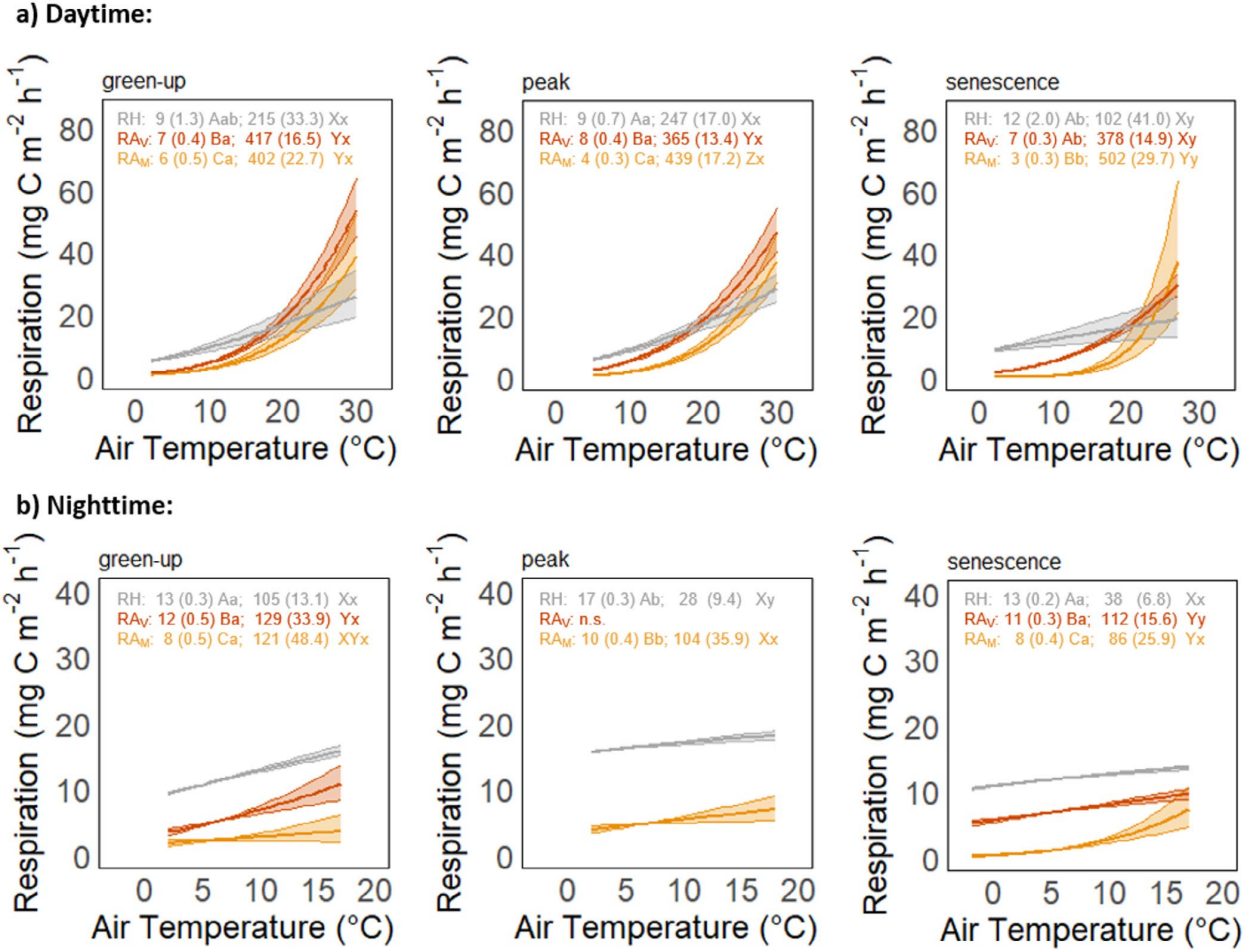
Response of heterotrophic respiration (RH, grey) and autotrophic respiration of vascular plant (RA_V_, orange) and *Sphagnum* mosses (RA_M_, yellow) to air temperature for (a) daytime and (b) nighttime (i.e., photosynthetic proton flux density < 20 μmol m^−2^ s^−1^) during the key phenophases of green‐up, peak season and senescence averaged over 2021–2023. Lines show significant regression fits based on the Lloyd and Taylor regression model (*p* < 0.05) with the shaded band representing the 95% confidence interval. The values in the panels display the estimated parameters *R*_10_ and *E*_0_ with standard error in brackets. Different capital and lowercase letters indicate significant differences between PFGs and among phenophases, respectively, based on the *z*‐test (*p* < 0.05).

The response of respiration to air temperature variations during day‐ and nighttime differed significantly between the key phenophases (Figure 6). Specifically, RA_M_ had lower base respiration (i.e., at 10°C, *R*_10_) values during day‐ and nighttime throughout all phenophases, compared to RA_v_ and RH. The *R*_10_ of RA_v_ was slightly lower compared to RH during day and nighttime, except during daytime senescence and nighttime during the peak season. Overall, there was limited variation across key phenophases for the daytime and nighttime *R*_10_ of RA_v_ and RA_M_. In comparison, day‐time temperature sensitivity (i.e., higher *E*_0_) of both PFGs was significantly higher during senescence compared to green‐up and peak season. Furthermore, daytime *E*_0_ of RA_M_ (*E*_0_ = 439; 502) was significantly higher than RA_V_ (*E*_0_ = 365; 378) in peak season and senescence. Nighttime *E*_0_ was considerably lower than daytime *E*_0_ across all phenophases for RA_M_, RA_V_ and RH. No significant differences in nighttime temperature sensitivity were found between RA_M_ and RA_V_.

## 4 Discussion

### 4.1 Seasonal Shifts in the Relative Importance of Vascular Plants and *Sphagnum* Mosses in Regulating Peatland NEE

In this study we present results from continuous automated chamber measurements of NEE and its components in a boreal peatland over three growing seasons. Our main findings reveal that vascular plants and *Sphagnum* mosses have distinct roles in regulating NEE via their varying contributions to RA and GPP across different key phenophases. This highlights the need to understand how the separate seasonal dynamics of these key PFGs, as well as their different responses to environmental drivers, will jointly regulate the carbon sink strength of northern peatlands in a future climate.

The growing season NEE of −64.2 to −62.6 g C m^−2^ measured during the study years (2021–2023) was within the range (−142 to 47 g C m^−2^) of long‐term EC estimates from the same site during 2001–2022 (Peichl et al. 2014; unpublished data) and similar to previous estimates from the same automated chamber system (−57.4 to −65.3 g C m^−2^ in 2015–2016; Järveoja et al. 2018). Our results suggest that variation in NEE across phenophases could be due to shifts in the relative importance of *Sphagnum* mosses and vascular plants in governing the underlying NEE components, i.e., GPP and ER. Specifically, we observed that *Sphagnum* mosses contributed more to GPP than vascular plants during green‐up and senescence, with the opposite being true during the peak season. Thus, seasonal variations in GPP may occur not only in response to weather patterns, but also due to shifts in the PFG's composition and phenology. A similar temporal shift in the relative importance of *Sphagnum* mosses and vascular GPP was previously indicated by monthly incubations of a peat mesocosm from a Finnish fen (Korrensalo et al. 2017). However, high temporal resolution data on PFG‐specific GPP from other peatlands are to our knowledge lacking, but critically needed to further evaluate if our findings are site‐specific or universal for boreal peatlands. At the growing season scale, the interannual variation in the relative importance of *Sphagnum* moss and vascular plant GPP was driven by variations in GPP_M_ (−82 g C m^−2^ in 2022 and −72 g C m^−2^ in 2023) whereas GPP_V_ remained similar (−70 g C m^−2^ in 2022 and −74 g C m^−2^ in 2023). However, the relative contribution of GPP_M_ and GPP_V_ may strongly vary across northern peatlands based on site‐specific vegetation characteristics. For instance, the contribution of GPP_M_ to GPP was 28% based on weekly to biweekly measurements in a boreal bog (Riutta et al. 2007) but 60% based on monthly mesocosm incubations from another boreal bog site (Korrensalo et al. 2017). Differences in the contribution of GPP_M_ and GPP_V_ to GPP might be dependent on nutrient status and vegetation composition, e.g., vascular plant green area index of 0.29 m^2^ m^−2^ in the study by Korrensalo et al. (2017) and 0.8 m^2^ m^−2^ in the study by Riutta et al. (2007). The temporal changes in the contribution are dependent on vegetation phenology and abiotic drivers. Thus, climate‐induced changes in vegetation composition and in the environmental conditions during distinct phenophases (e.g., summer drought) could largely alter the separate contributions from moss and plant production, with potentially far‐reaching implications for the peatland C cycle.

Our results further demonstrate considerable differences in the contribution of *Sphagnum* mosses and vascular plants to ER. Specifically, we observed consistently higher contribution (2–17 times) and variability of RA_V_ compared to RA_M_ which might be explained by the physiological and structural differences between these PFGs, namely the greater growth and maintenance respiration of vascular plants due to higher biomass production. It is noteworthy that the partitioning of ER fluxes into RA and RH is based on the assumption that RH derived from trenched vegetation removal plots represents the actual heterotrophic respiration in the vegetated plots. However, RH from vegetation removal plots only represents fluxes associated with the decomposition of old peat, while it does not capture the microbial respiration of recent root exudates. Thus, RH estimates from these experimental plots might be underestimated, which in turn would result in overestimated RA_M_ as well as NPP as the latter are derived from the differences between ER and NEE fluxes from vegetated and RH from vegetation‐removal plots. Furthermore, our partitioning approach to derive RA_M_ from the relatively small difference between total respiration from the moss plots and RH reached its limits during cold and wet conditions in green‐up and senescence. During these periods, RA_M_ was too low to create a difference in respiration from moss and RH plots. The comparison of the growing season sums revealed that RA_V_ and RA_M_ contributed 38%–40% and 3%–11% of ER, respectively. Similar contributions of RA_V_ to ER in the range of 35%–57% have been previously reported based on ^14^C pulse labeling (Crow and Wieder 2005) and bi‐weekly daytime chamber flux measurements in a boreal bog (Riutta et al. 2007). The contribution of RA_V_ to growing season RA (78%–93%) at our site is at the upper end of the range (33%–85%) previously reported by studies based on weekly to monthly manual chamber measurements across other boreal and high altitude peatlands (Gavazov et al. 2018; Rankin et al. 2022; Riutta et al. 2007). Thus, the relative importance of RA_V_ and RA_M_ seems to be highly dependent on vegetation composition and changes in abiotic conditions throughout the growing season for peatlands.

NPP is a crucial flux in the peatland C balance as it essentially determines the net C input in the form of organic matter production (Gorham 1991). However, moss NPP is commonly estimated at the growing season scale only based on the brushwire method, while NPP_V_ is often limited to aboveground components assessed by repeated inventory in bi‐weekly or less frequent intervals (Järveoja et al. 2018; Laine et al. 2012; Leppälä et al. 2008; Moore et al. 2002; Riutta et al. 2007). Compared to those methods, our chamber‐based estimates of daily NPP_M_ and NPP_V_ revealed the considerable daily to seasonal variations in the contribution of NPP_M_ to NPP. Surprisingly, during the peak season, moss productivity accounted for 50%–60% of total NPP, while their contribution to GPP was lower (44%–48%), suggesting a relatively greater efficiency of *Sphagnum* mosses to convert assimilated carbon into biomass, relative to vascular plants (Street et al. 2013). It is further noteworthy that the ratio of NPP_M_:GPP_M_ > 1 observed during senescence is physiologically not possible. This unrealistic estimate was likely due to methodological limitations in the partitioning of the *Sphagnum* moss fluxes during senescence, during which productivity levels are low. The growing season sum of NPP_M_ (−63 to −79 g C m^−2^) at our site was well in the range reported from other boreal poor fens (−27 to −287 g C m^−2^ year^−1^) (Moore et al. 2002). Our chamber‐based NPP estimates were also in close agreement with concurrent brushwire estimates in 2022 (−56 g C m^−2^). Thus, our flux partitioning approach delivered reasonable estimates of moss NPP while also providing valuable insights into the seasonal dynamics of NPP_V_ and NPP_M_. The observed variation of contribution of NPP_V_ and NPP_M_ to NPP between phenophases highlights the need for a deeper understanding of the seasonal patterns of PFG specific productivity to improve estimates of NPP and its response to global change impacts.

### 4.2 Distinct Environmental Drivers for Vascular Plant and *Sphagnum* Moss Production and Respiration Fluxes

Our stepwise GLM analysis revealed an alternating dominance of biotic and abiotc factors regulating GPP for both *Sphagnum* mosses and vascular plants across the key phenophases. Furthermore, while plant phenology was the key driver for GPP_V_ and GPP_M_ during green‐up, the effect size was larger for vascular plants, indicating a stronger link between vegetation phenology and vascular plant productivity than GPP_M_ in the early growing season. In contrast, we noted that radiation was the driver for GPP_V_ and GPP_M_ during peak season and senescence. This implies that increased cloudiness in response to climate change would affect GPP_V_ and GPP_M_ more during the later than the early growing season. In addition, our results suggest that GPP_V_ is more sensitive to changes in radiation during peak season and senescence compared to GPP_M_. This observation also aligns with the higher maximum photosynthetic capacity (i.e., *A*_max_) of vascular plants noted during peak season and senescence compared to green‐up. Furthermore, the importance of vascular plants for regulating maximum CO_2_ assimilation was also evident from the higher seasonal variation in their *A*_max_, which was about to twice as large for vascular plants (2.2‐ to 3.9‐fold) than for *Sphagnum* mosses (1.5‐ to 2.4‐fold). Altogether, this highlights that despite the important contribution of mosses to total GPP, the day‐to‐day variations in peatland GPP are particularly dependent on vascular plant productivity and radiation supply in the later growing season (peak and senescence). In contrast, the higher initial light use efficiency noted for *Sphagnum* mosses during green‐up and senescence implies that *Sphagnum* mosses can outperform vascular plants under limited light conditions. This may explain the dominance of GPP_M_ during senescence during which light levels rapidly decrease in high latitude regions. Higher light use efficiency of *Sphagnum* mosses has been previously reported based on monthly measurement campaigns averaged at the growing season scale (Korrensalo et al. 2016; Leppälä et al. 2008; Peichl et al. 2018). The high‐temporal resolution data from our continuous chamber measurements further advance this understanding by facilitating an assessment of the seasonal variation of *A*_max_ and *α* of vascular plants and *Sphagnum* mosses at sub‐seasonal scales. Altogether, our results highlight that vegetation composition and phenology strongly modulate the response of the peatland GPP to environmental conditions.

While GPP was regulated exclusively by gcc and PPFD, our stepwise GLM revealed that RA_M_ and RA_V_ were governed by multiple factors and their interactions. It is further noteworthy that the effect sizes of the key controls (i.e., *T*_a_, WTL, PPFD, gcc) on RA_M_ varied considerably more across phenophases than for RA_V_. This suggests that RA_M_ is regulated by a more complex driver network which likely explains its greater variation to changes in environmental conditions, relative to RA_V_. For instance, WTL had a strong negative effect on RA_M_ during green‐up (enhanced by interaction with PPFD) that is possibly related to reduced moss growth and physiological stress under fully saturated and oxygen‐limited conditions (Strack et al. 2006). The switch of PFFD from having a negative effect in green‐up to a positive effect in peak season further exemplifies the complex controls on RA_M_. In contrast, air temperature had a consistently positive effect on RA_M_. Altogether, these results highlight the higher sensitivity of RA_M_ to a changing climate compared to RA_V_, demonstrating that vegetation composition may considerably modulate RA dynamics.

Our results further revealed distinct responses of RA_M_ and RA_V_ to diel changes in air temperature, suggesting higher base respiration rate (at 10°C) during nighttime and higher temperature sensitivity (i.e., the *E*_0_ parameter) during daytime for both PFGs. The diel divergence of temperature sensitivity has been previously shown for RH and RA at the same peatland site (Järveoja et al. 2020) and globally for growing season ER in diverse terrestrial ecosystems (Li et al. 2024). This diel divergence of RA might be due to different contributions from plant growth versus maintenance respiration (Thornley 1970), contrasting abiotic drivers and/or a lag in the above‐ to belowground allocation of recent photosynthates (Bahn et al. 2009; Järveoja et al. 2020; Phillips et al. 2010; Vargas and Allen 2008). Here, we advance this understanding by providing insights into the separate responses of respiration from *Sphagnum* mosses and vascular plants. Specifically, our findings highlight considerable seasonal differences in daytime temperature sensitivity, with RA_M_ being more sensitive than RA_V_ during peak season and senescence, while no differences between PFGs were noted during nighttime. Combined with the observation that base respiration at 10°C was higher for RA_V_ compared to RA_M_ across all phenophases, this leads to strongly differing temperature responses of the PFG‐specific RA components. These findings are of importance given that contrasting trends in day‐ and nighttime temperature regimes (i.e., mean, maxima, minima) have been noted in the boreal biome in response to climate change (Deng et al. 2019; Peng et al. 2013; Tan et al. 2015). For instance, given the higher base respiration and lack of PFG differences during night found in our study, the faster increase of nighttime temperatures relative to daytime temperatures observed in the boreal region (Tan et al. 2015) may enhance nighttime RA losses irrespectively of vegetation composition, potentially leading to a reduced peatland C sink strength. Thus, an improved understanding of diel respiration dynamics and responses to climate warming is required.

### 4.3 Implications for the Peatland Carbon Cycle in a Changing Climate

Our findings reveal that the responses of PFG‐specific production and respiration to environmental changes are strongly dependent on the seasonal timing of weather patterns, extreme events and climatic shifts. For instance, our results imply that a climate‐induced increase in cloud cover during the mid and late growing season would affect GPP of *Sphagnum* mosses and especially vascular plants more strongly than in the early season. Furthermore, the greater sensitivity of *Sphagnum* moss respiration to environmental changes, including increased RA_M_ due to the lowering of WTL during spring and summer droughts as well as an increased daytime temperature response in summer and autumn, underscores the vulnerability of this PFG to future climatic changes. Altogether, our results provide important empirical evidence to help explain the complex responses of the peatland NEE to environmental conditions observed across the boreal biome (Helbig et al. 2022).

Our study further contributes important insights to the discussion about whether an extended growing season length in a future warmer climate will result in increased net CO_2_ uptake (Antala et al. 2022; Helbig et al. 2022). Specifically, our results suggest that an earlier onset of the growing season will promote the productivity of *Sphagnum* moss rather than that of vascular plants, as *Sphagnum* moss growth may start directly after snow melt and mean daily air temperatures exceed 0°C (Moore et al. 2006). Similarly, the later onset of snowfall and soil frost in a warmer climate would favour higher productivity of *Sphagnum* mosses compared to vascular plants during the extended autumn period, given the greater efficiency of mosses in utilizing low light conditions. Ultimately, the response of the net CO_2_ balance to an extended growing season will also depend on the consequences for RH (Parmentier et al. 2011; Piao et al. 2008; Tang et al. 2022). A more elaborated analysis of potential impacts of climate change scenarios on the peatland NEE would be highly relevant.

The complex interplay of environmental factors with vegetation composition and phenology in regulating NEE also poses a challenge for process‐based model predictions of peatland C cycle responses to climate change (Richardson et al. 2012). At present, peatland and vegetation models (e.g., PCARS, PEAT‐CLSM, LPJ, ORCHIDEE‐PEAT, ELM and CoupModel) commonly include phenology explicitly using the growing degree approach or by incorporating its effect on GPP through changes in leaf area index (LPJ, ORCHIDEE‐PEAT, ELM, PCARS, MWM, CoupModel) (Chaudhary et al. 2017; Frolking et al. 2002; He et al. 2025; Meng et al. 2021; Qiu et al. 2018; St‐Hilaire et al. 2010). However, these approaches require PFG‐specific and likely even species‐specific thresholds for growing degree days or leaf area index relationships with plant functioning. This knowledge is currently limited for the various vascular plant species, and commonly lacking for *Sphagnum* mosses, except for recent advances in adding *Sphagnum* specific thresholds in CoupModel and ORCHIDEE (He et al. 2025; Liu et al. 2025). Furthermore, PFG‐specific responses to environmental conditions and their consequences for regulating production and respiration fluxes in different phenophases is not implemented in state‐of‐the‐art peatland models so far. Our study therefore highlights the need for incorporating detailed information for temporal dynamics and controls of PFG‐specific net CO_2_ component fluxes into current process‐based models to improve our ability to accurately predict the response of the peatland C cycle to future global changes.

## Author Contributions

**Antonia Hartmann:** conceptualization, data curation, formal analysis, investigation, methodology, visualization, writing – original draft. **Kyohsuke Hikino:** data curation, writing – review and editing. **Lukas Guth:** data curation, writing – review and editing. **Gillian Simpson:** data curation, writing – review and editing. **Järvi Järveoja:** data curation, methodology, writing – review and editing. **Mats B. Nilsson:** methodology, resources, writing – review and editing. **Matthias Peichl:** conceptualization, data curation, funding acquisition, investigation, methodology, project administration, resources, supervision, writing – review and editing.

## Funding

This work was supported by Swedish Research Council (2019‐04676) and Kempe Foundation (JCK‐3156 and JCSMK23‐0221).

## Conflicts of Interest

The authors declare no conflicts of interest.

## Supporting information


**Figure S1:** Pictures of the closed shading umbrella during dark measurements on vegetated plots.
**Figure S2:** Filtering criteria for discarding poor quality fluxes.
**Figure S3:** Treatment‐specific nighttime flux correction.
**Figure S4:** Measured and modelled chamber CO_2_ fluxes.
**Table S1:** Total number of potential chamber measurements per growing season, amount of data removed during quality‐control filtering (%) and coefficient of determination (*R* ^2^) of predicted to gap‐filled fluxes for holdout sets during 10‐fold cross validation using XGBoost per chamber.
**Table S2:** Maximum green vascular plant biomass (g DW m^‐2^) within the automated natural chamber plots per growing season 2021–2023.
**Table S3:** Results of stepwise general linear model for abiotic (PPFD, photosynthetic photon flux density; *T*_a_, air temperature; WTL, water table level) and biotic factors (gcc, green chromatic coordinate used as a proxy for greenness) explaining daily variation of gross primary production (GPP) and autotrophic respiration (RA) of *Sphagnum* mosses (_M_) and vascular plants (_V_). Significance levels are denoted with asteriks * (*p*‐value < 0.05), ** (< 0.01) and *** (< 0.001).

## Data Availability

The data supporting the findings of this study are openly available in the Dryad digital repository at https://doi.org/10.5061/dryad.pvmcvdnz8.

## References

[gcb70834-bib-0001] Antala, M. , R. Juszczak , C. van der Tol , and A. Rastogi . 2022. “Impact of Climate Change‐Induced Alterations in Peatland Vegetation Phenology and Composition on Carbon Balance.” Science of the Total Environment 827: 154294. 10.1016/j.scitotenv.2022.154294. 35247401

[gcb70834-bib-0002] Armstrong, A. , S. Waldron , N. J. Ostle , H. Richardson , and J. Whitaker . 2015. “Biotic and Abiotic Factors Interact to Regulate Northern Peatland Carbon Cycling.” Ecosystems 18, no. 8: 1395–1409. 10.1007/s10021-015-9907-4.

[gcb70834-bib-0003] Bahn, M. , M. Schmitt , R. Siegwolf , A. Richter , and N. Brüggemann . 2009. “Does Photosynthesis Affect Grassland Soil‐Respired CO_2_ and Its Carbon Isotope Composition on a Diurnal Timescale?” New Phytologist 182, no. 2: 451–460. 10.1111/j.1469-8137.2008.02755.x. 19220762 PMC2950940

[gcb70834-bib-0004] Bond‐Lamberty, B. , D. Bronson , E. Bladyka , and S. T. Gower . 2011. “A Comparison of Trenched Plot Techniques for Partitioning Soil Respiration.” Soil Biology and Biochemistry 43, no. 10: 2108–2114. 10.1016/j.soilbio.2011.06.011.

[gcb70834-bib-0005] Brændholt, A. , K. Steenberg Larsen , A. Ibrom , and K. Pilegaard . 2017. “Overestimation of Closed‐Chamber Soil CO_2_ Effluxes at Low Atmospheric Turbulence.” Biogeosciences 14, no. 6: 1603–1616. 10.5194/bg-14-1603-2017.

[gcb70834-bib-0006] Bubier, J. , P. Crill , A. Mosedale , S. Frolking , and E. Linder . 2003. “Peatland Responses to Varying Interannual Moisture Conditions as Measured by Automatic CO_2_ Chambers.” Global Biogeochemical Cycles 17, no. 2: 1066. 10.1029/2002GB001946.

[gcb70834-bib-0007] Bubier, J. L. , P. M. Crill , T. R. Moore , K. Savage , and R. K. Varner . 1998. “Seasonal Patterns and Controls on Net Ecosystem CO_2_ Exchange in a Boreal Peatland Complex.” Global Biogeochemical Cycles 12, no. 4: 703–714. 10.1029/98GB02426.

[gcb70834-bib-0008] Calvin, K. , D. Dasgupta , G. Krinner , et al. 2023. “IPCC, 2023: Climate Change 2023: Synthesis Report.” In Contribution of Working Groups I, II and III to the Sixth Assessment Report of the Intergovernmental Panel on Climate Change, edited by Core Writing Team , H. Lee , and J. Romero . IPCC. 10.59327/IPCC/AR6-9789291691647.

[gcb70834-bib-0009] Chaudhary, N. , P. A. Miller , and B. Smith . 2017. “Modelling Holocene Peatland Dynamics With an Individual‐Based Dynamic Vegetation Model.” Biogeosciences 14, no. 10: 2571–2596. 10.5194/bg-14-2571-2017.

[gcb70834-bib-0010] Christensen, J. H. , and O. B. Christensen . 2007. “A Summary of the PRUDENCE Model Projections of Changes in European Climate by the End of This Century.” Climatic Change 81, no. S1: 7–30. 10.1007/s10584-006-9210-7.

[gcb70834-bib-0011] Crow, S. , and R. Wieder . 2005. “Sources of CO_2_ Emission From a Northern Peatland: Root Respiration, Exudation, and Decomposition.” Ecology 86: 1825–1834. 10.1890/04-1575.

[gcb70834-bib-0012] Deng, G. , H. Zhang , X. Guo , et al. 2019. “Asymmetric Effects of Daytime and Nighttime Warming on Boreal Forest Spring Phenology.” Remote Sensing 11, no. 14: 14. 10.3390/rs11141651.

[gcb70834-bib-0013] Dinsmore, K. J. , M. F. Billett , U. M. Skiba , R. M. Rees , J. Drewer , and C. Helfter . 2010. “Role of the Aquatic Pathway in the Carbon and Greenhouse Gas Budgets of a Peatland Catchment.” Global Change Biology 16, no. 10: 2750–2762. 10.1111/j.1365-2486.2009.02119.x.

[gcb70834-bib-0014] Dise, N. B. 2009. “Peatland Response to Global Change.” Science 326, no. 5954: 810–811. 10.1126/science.1174268. 19892972

[gcb70834-bib-0015] Frolking, S. , N. T. Roulet , T. R. Moore , P. M. Lafleur , J. L. Bubier , and P. M. Crill . 2002. “Modeling Seasonal to Annual Carbon Balance of Mer Bleue Bog, Ontario, Canada.” Global Biogeochemical Cycles 16, no. 3: 4‐1–4‐21. 10.1029/2001GB001457.

[gcb70834-bib-0016] Gavazov, K. , R. Albrecht , A. Buttler , et al. 2018. “Vascular Plant‐Mediated Controls on Atmospheric Carbon Assimilation and Peat Carbon Decomposition Under Climate Change.” Global Change Biology 24, no. 9: 3911–3921. 10.1111/gcb.14140. 29569798

[gcb70834-bib-0017] Gažovič, M. , I. Forbrich , D. F. Jager , L. Kutzbach , C. Wille , and M. Wilmking . 2013. “Hydrology‐Driven Ecosystem Respiration Determines the Carbon Balance of a Boreal Peatland.” Science of the Total Environment 463–464: 675–682. 10.1016/j.scitotenv.2013.06.077. 23845859

[gcb70834-bib-0018] Gielen, B. , M. Acosta , N. Altimir , et al. 2018. “Ancillary Vegetation Measurements at ICOS Ecosystem Stations.” International Agrophysics 32, no. 4: 645–664. 10.1515/intag-2017-0048.

[gcb70834-bib-0019] Gorham, E. 1991. “Northern Peatlands: Role in the Carbon Cycle and Probable Responses to Climatic Warming.” Ecological Applications 1, no. 2: 182–195. 10.2307/1941811. 27755660

[gcb70834-bib-0020] Görres, C.‐M. , C. Kammann , and R. Ceulemans . 2016. “Automation of Soil Flux Chamber Measurements: Potentials and Pitfalls.” Biogeosciences 13, no. 6: 1949–1966. 10.5194/bg-13-1949-2016.

[gcb70834-bib-0021] Goulden, M. L. , and P. M. Crill . 1997. “Automated Measurements of CO_2_ Exchange at the Moss Surface of a Black Spruce Forest.” Tree Physiology 17, no. 8–9: 537–542. 10.1093/treephys/17.8-9.537. 14759826

[gcb70834-bib-0022] Grace, J. 2004. “Understanding and Managing the Global Carbon Cycle.” Journal of Ecology 92, no. 2: 189–202. 10.1111/j.0022-0477.2004.00874.x.

[gcb70834-bib-0023] Gu, L. , W. M. Post , D. D. Baldocchi , et al. 2009. “Characterizing the Seasonal Dynamics of Plant Community Photosynthesis Across a Range of Vegetation Types.” In Phenology of Ecosystem Processes: Applications in Global Change Research, edited by A. Noormets , 35–58. Springer. 10.1007/978-1-4419-0026-5_2.

[gcb70834-bib-0024] Gunnarsson, U. 2005. “Global Patterns of Sphagnum Productivity.” Journal of Bryology 27, no. 3: 269–279. 10.1179/174328205X70029.

[gcb70834-bib-0025] He, H. , T. Moore , P. Lafleur , et al. 2025. “Spring Phenology in Photosynthesis Control and Modeling for a Temperate Bog.” Frontiers in Environmental Science 13. 10.3389/fenvs.2025.1548578.

[gcb70834-bib-0026] Helbig, M. , T. Živković , P. Alekseychik , et al. 2022. “Warming Response of Peatland CO_2_ Sink Is Sensitive to Seasonality in Warming Trends.” Nature Climate Change 12, no. 8: 743–749. 10.1038/s41558-022-01428-z.

[gcb70834-bib-0027] Helfter, C. , C. Campbell , K. J. Dinsmore , et al. 2015. “Drivers of Long‐Term Variability in CO_2_ Net Ecosystem Exchange in a Temperate Peatland.” Biogeosciences 12, no. 6: 1799–1811. 10.5194/bg-12-1799-2015.

[gcb70834-bib-0028] Järveoja, J. , M. B. Nilsson , P. M. Crill , and M. Peichl . 2020. “Bimodal Diel Pattern in Peatland Ecosystem Respiration Rebuts Uniform Temperature Response.” Nature Communications 11, no. 1: 4255. 10.1038/s41467-020-18027-1. PMC7449960 32848144

[gcb70834-bib-0029] Järveoja, J. , M. B. Nilsson , M. Gažovič , P. M. Crill , and M. Peichl . 2018. “Partitioning of the Net CO _2_ Exchange Using an Automated Chamber System Reveals Plant Phenology as Key Control of Production and Respiration Fluxes in a Boreal Peatland.” Global Change Biology 24, no. 8: 3436–3451. 10.1111/gcb.14292. 29710420

[gcb70834-bib-0030] Koebsch, F. , O. Sonnentag , J. Järveoja , et al. 2020. “Refining the Role of Phenology in Regulating Gross Ecosystem Productivity Across European Peatlands.” Global Change Biology 26, no. 2: 876–887. 10.1111/gcb.14905. 31686431

[gcb70834-bib-0031] Koehler, A.‐K. , M. Sottocornola , and G. Kiely . 2011. “How Strong Is the Current Carbon Sequestration of an Atlantic Blanket Bog?” Global Change Biology 17, no. 1: 309–319. 10.1111/j.1365-2486.2010.02180.x.

[gcb70834-bib-0032] Korrensalo, A. , P. Alekseychik , T. Hájek , et al. 2017. “Species‐Specific Temporal Variation in Photosynthesis as a Moderator of Peatland Carbon Sequestration.” Biogeosciences 14, no. 2: 257–269. 10.5194/bg-14-257-2017.

[gcb70834-bib-0033] Korrensalo, A. , T. Hájek , T. Vesala , L. Mehtätalo , and E.‐S. Tuittila . 2016. “Variation in Photosynthetic Properties Among Bog Plants.” Botany 94, no. 12: 1127–1139. 10.1139/cjb-2016-0117.

[gcb70834-bib-0034] Kross, A. , J. W. Seaquist , and N. T. Roulet . 2016. “Light Use Efficiency of Peatlands: Variability and Suitability for Modeling Ecosystem Production.” Remote Sensing of Environment 183: 239–249. 10.1016/j.rse.2016.05.004.

[gcb70834-bib-0035] Lai, D. Y. F. , N. T. Roulet , E. R. Humphreys , T. R. Moore , and M. Dalva . 2012. “The Effect of Atmospheric Turbulence and Chamber Deployment Period on Autochamber CO_2_ and CH_4_ Flux Measurements in an Ombrotrophic Peatland.” Biogeosciences 9, no. 8: 3305–3322. 10.5194/bg-9-3305-2012.

[gcb70834-bib-0036] Laine, A. M. , J. Bubier , T. Riutta , et al. 2012. “Abundance and Composition of Plant Biomass as Potential Controls for Mire Net Ecosytem CO_2_ Exchange.” Botany 90, no. 1: 63–74. 10.1139/b11-068.

[gcb70834-bib-0037] Laine, A. M. , A. Korrensalo , and E.‐S. Tuittila . 2022. “Plant Functional Traits Play the Second Fiddle to Plant Functional Types in Explaining Peatland CO_2_ and CH_4_ Gas Exchange.” Science of the Total Environment 834: 155352. 10.1016/j.scitotenv.2022.155352. 35460776

[gcb70834-bib-0038] Leppälä, M. , K. Kukko‐Oja , J. Laine , and E.‐S. Tuittila . 2008. “Seasonal Dynamics of CO_2_ Exchange During Primary Succession of Boreal Mires as Controlled by Phenology of Plants.” Écoscience 15, no. 4: 460–471. 10.2980/15-4-3142.

[gcb70834-bib-0039] Li, N. , G. Zhou , M. Krishna , et al. 2024. “Warming Diminishes the Day–Night Discrepancy in the Apparent Temperature Sensitivity of Ecosystem Respiration.” Plants 13, no. 23. 10.3390/plants13233321. PMC11644270 39683114

[gcb70834-bib-0040] Lieth, H. 1974. Phenology and Seasonality Modeling. Vol. 8. Springer. 10.1007/978-3-642-51863-8.

[gcb70834-bib-0041] Linkosalmi, M. , M. Aurela , J.‐P. Tuovinen , et al. 2016. “Digital Photography for Assessing the Link Between Vegetation Phenology and CO_2_ Exchange in Two Contrasting Northern Ecosystems.” Geoscientific Instrumentation, Methods and Data Systems 5, no. 2: 417–426. 10.5194/gi-5-417-2016.

[gcb70834-bib-0042] Liu, L. , C. Qiu , Y. Xi , et al. 2025. “Assessing CO_2_ Fluxes for European Peatlands in ORCHIDEE‐PEAT With Multiple Plant Functional Types.” Journal of Advances in Modeling Earth Systems 17, no. 6: e2025MS004940. 10.1029/2025MS004940.

[gcb70834-bib-0043] Lloyd, J. , and J. A. Taylor . 1994. “On the Temperature Dependence of Soil Respiration.” Functional Ecology 8, no. 3: 315–323. 10.2307/2389824.

[gcb70834-bib-0044] Loisel, J. , A. V. Gallego‐Sala , and Z. Yu . 2012. “Global‐Scale Pattern of Peatland *Sphagnum* Growth Driven by Photosynthetically Active Radiation and Growing Season Length.” Biogeosciences 9, no. 7: 2737–2746. 10.5194/bg-9-2737-2012.

[gcb70834-bib-0045] Mastný, J. , J. Bárta , E. Kaštovská , and T. Picek . 2021. “Decomposition of Peatland DOC Affected by Root Exudates Is Driven by Specific r and K Strategic Bacterial Taxa.” Scientific Reports 11, no. 1: 18677. 10.1038/s41598-021-97698-2. 34548501 PMC8455546

[gcb70834-bib-0046] Meng, L. , J. Mao , D. M. Ricciuto , et al. 2021. “Evaluation and Modification of ELM Seasonal Deciduous Phenology Against Observations in a Southern Boreal Peatland Forest.” Agricultural and Forest Meteorology 308–309: 108556. 10.1016/j.agrformet.2021.108556.

[gcb70834-bib-0047] Moore, T. R. , J. L. Bubier , S. E. Frolking , P. M. Lafleur , and N. T. Roulet . 2002. “Plant Biomass and Production and CO_2_ Exchange in an Ombrotrophic Bog.” Journal of Ecology 90, no. 1: 25–36. 10.1046/j.0022-0477.2001.00633.x.

[gcb70834-bib-0048] Moore, T. R. , P. M. Lafleur , D. M. I. Poon , B. W. Heumann , J. W. Seaquist , and N. T. Roulet . 2006. “Spring Photosynthesis in a Cool Temperate Bog.” Global Change Biology 12, no. 12: 2323–2335. 10.1111/j.1365-2486.2006.01247.x.

[gcb70834-bib-0049] Nielsen, C. S. , N. J. Hasselquist , M. B. Nilsson , M. Öquist , J. Järveoja , and M. Peichl . 2019. “A Novel Approach for High‐Frequency In‐Situ Quantification of Methane Oxidation in Peatlands.” Soil Systems 3, no. 1. 10.3390/soilsystems3010004.

[gcb70834-bib-0050] Nilsson, M. , J. Sagerfors , I. Buffam , et al. 2008. “Contemporary Carbon Accumulation in a Boreal Oligotrophic Minerogenic Mire – A Significant Sink After Accounting for All C‐Fluxes.” Global Change Biology 14, no. 10: 2317–2332. 10.1111/j.1365-2486.2008.01654.x.

[gcb70834-bib-0051] Nilsson, M. B. , M. Peichl , P. Marklund , et al. 2025. “*ETC L2 Meteo From Degero* (11676/mke1xZgsnTVrG1EAzyZT1Dxi)” [Data Set]. https://hdl.handle.net/11676/mke1xZgsnTVrG1EAzyZT1Dxi.

[gcb70834-bib-0052] Noumonvi, K. D. , A. M. Ågren , J. L. Ratcliffe , et al. 2023. “The Kulbäcksliden Research Infrastructure: A Unique Setting for Northern Peatland Studies.” Frontiers in Earth Science 11: 1194749. 10.3389/feart.2023.1194749.

[gcb70834-bib-0053] Olefeldt, D. , N. T. Roulet , O. Bergeron , P. Crill , K. Bäckstrand , and T. R. Christensen . 2012. “Net Carbon Accumulation of a High‐Latitude Permafrost Palsa Mire Similar to Permafrost‐Free Peatlands.” Geophysical Research Letters 39, no. 3: L03501. 10.1029/2011GL050355.

[gcb70834-bib-0054] Page, S. E. , and A. J. Baird . 2016. “Peatlands and Global Change: Response and Resilience.” Annual Review of Environment and Resources 41, no. 1: 35–57. 10.1146/annurev-environ-110615-085520.

[gcb70834-bib-0055] Parmentier, F. J. W. , M. K. van der Molen , J. van Huissteden , et al. 2011. “Longer Growing Seasons Do Not Increase Net Carbon Uptake in the Northeastern Siberian Tundra.” Journal of Geophysical Research: Biogeosciences 116, no. G4: G04013. 10.1029/2011JG001653.

[gcb70834-bib-0056] Peichl, M. , M. Gažovič , I. Vermeij , et al. 2018. “Peatland Vegetation Composition and Phenology Drive the Seasonal Trajectory of Maximum Gross Primary Production.” Scientific Reports 8, no. 1: 8012. 10.1038/s41598-018-26147-4. 29789673 PMC5964230

[gcb70834-bib-0057] Peichl, M. , M. Öquist , M. Ottosson Löfvenius , et al. 2014. “A 12‐Year Record Reveals Pre‐Growing Season Temperature and Water Table Level Threshold Effects on the Net Carbon Dioxide Exchange in a Boreal Fen.” Environmental Research Letters 9, no. 5: 055006. 10.1088/1748-9326/9/5/055006.

[gcb70834-bib-0058] Peichl, M. , O. Sonnentag , and M. B. Nilsson . 2015. “Bringing Color Into the Picture: Using Digital Repeat Photography to Investigate Phenology Controls of the Carbon Dioxide Exchange in a Boreal Mire.” Ecosystems 18, no. 1: 115–131. 10.1007/s10021-014-9815-z.

[gcb70834-bib-0059] Peng, S. , S. Piao , P. Ciais , et al. 2013. “Asymmetric Effects of Daytime and Night‐Time Warming on Northern Hemisphere Vegetation.” Nature 501, no. 7465: 88–92. 10.1038/nature12434. 24005415

[gcb70834-bib-0060] Phillips, S. C. , R. K. Varner , S. Frolking , et al. 2010. “Interannual, Seasonal, and Diel Variation in Soil Respiration Relative to Ecosystem Respiration at a Wetland to Upland Slope at Harvard Forest.” Journal of Geophysical Research: Biogeosciences 115, no. G2: G02019. 10.1029/2008JG000858.

[gcb70834-bib-0061] Piao, S. , P. Ciais , P. Friedlingstein , et al. 2008. “Net Carbon Dioxide Losses of Northern Ecosystems in Response to Autumn Warming.” Nature 451, no. 7174: 49–52. 10.1038/nature06444. 18172494

[gcb70834-bib-0062] Qiu, C. , D. Zhu , P. Ciais , et al. 2018. “ORCHIDEE‐PEAT (Revision 4596), a Model for Northern Peatland CO_2_, Water, and Energy Fluxes on Daily to Annual Scales.” Geoscientific Model Development 11, no. 2: 497–519. 10.5194/gmd-11-497-2018.

[gcb70834-bib-0063] Rankin, T. E. , N. T. Roulet , and T. R. Moore . 2022. “Controls on Autotrophic and Heterotrophic Respiration in an Ombrotrophic Bog.” Biogeosciences 19, no. 13: 3285–3303. 10.5194/bg-19-3285-2022.

[gcb70834-bib-0064] Richardson, A. D. , R. S. Anderson , M. A. Arain , et al. 2012. “Terrestrial Biosphere Models Need Better Representation of Vegetation Phenology: Results From the North American Carbon Program Site Synthesis.” Global Change Biology 18, no. 2: 566–584. 10.1111/j.1365-2486.2011.02562.x.

[gcb70834-bib-0065] Riederer, M. , A. Serafimovich , and T. Foken . 2014. “Net Ecosystem CO_2_ Exchange Measurements by the Closed Chamber Method and the Eddy Covariance Technique and Their Dependence on Atmospheric Conditions.” Atmospheric Measurement Techniques 7, no. 4: 1057–1064. 10.5194/amt-7-1057-2014.

[gcb70834-bib-0066] Riutta, T. , J. Laine , and E.‐S. Tuittila . 2007. “Sensitivity of CO_2_ Exchange of Fen Ecosystem Components to Water Level Variation.” Ecosystems 10, no. 5: 718–733. 10.1007/s10021-007-9046-7.

[gcb70834-bib-0067] Roulet, N. , P. Lafleur , P. Richard , T. Moore , E. Humphreys , and J. Bubier . 2007. “Contemporary Carbon Balance an Late Holocene Carbon Accumulation in a Northern Peatland.” Global Change Biology 13: 397–411. 10.1111/j.1365-2486.2006.01292.x.

[gcb70834-bib-0068] Rydin, H. , and J. K. Jeglum . 2013. The Biology of Peatlands. Oxford University Press. 10.1093/acprof:osobl/9780199602995.001.0001.

[gcb70834-bib-0069] Savage, K. , E. A. Davidson , and J. Tang . 2013. “Diel Patterns of Autotrophic and Heterotrophic Respiration Among Phenological Stages.” Global Change Biology 19, no. 4: 1151–1159. 10.1111/gcb.12108. 23504892

[gcb70834-bib-0070] Schneider, J. , L. Kutzbach , S. Schulz , and M. Wilmking . 2009. “Overestimation of CO_2_ Respiration Fluxes by the Closed Chamber Method in Low‐Turbulence Nighttime Conditions.” Journal of Geophysical Research: Biogeosciences 114, no. G3: G03005. 10.1029/2008JG000909.

[gcb70834-bib-0071] St‐Hilaire, F. , J. Wu , N. T. Roulet , et al. 2010. “McGill Wetland Model: Evaluation of a Peatland Carbon Simulator Developed for Global Assessments.” Biogeosciences 7, no. 11: 3517–3530. 10.5194/bg-7-3517-2010.

[gcb70834-bib-0072] Strachan, I. B. , L. Pelletier , and M.‐C. Bonneville . 2016. “Inter‐Annual Variability in Water Table Depth Controls Net Ecosystem Carbon Dioxide Exchange in a Boreal Bog.” Biogeochemistry 127, no. 1: 99–111. 10.1007/s10533-015-0170-8.

[gcb70834-bib-0073] Strack, M. , J. M. Waddington , L. Rochefort , and E.‐S. Tuittila . 2006. “Response of Vegetation and Net Ecosystem Carbon Dioxide Exchange at Different Peatland Microforms Following Water Table Drawdown.” Journal of Geophysical Research: Biogeosciences 111, no. G2: G02006. 10.1029/2005JG000145.

[gcb70834-bib-0074] Street, L. E. , J. Subke , M. Sommerkorn , et al. 2013. “The Role of Mosses in Carbon Uptake and Partitioning in Arctic Vegetation.” New Phytologist 199, no. 1: 163–175. 10.1111/nph.12285. 23614757

[gcb70834-bib-0075] Tan, J. , S. Piao , A. Chen , et al. 2015. “Seasonally Different Response of Photosynthetic Activity to Daytime and Night‐Time Warming in the Northern Hemisphere.” Global Change Biology 21, no. 1: 377–387. 10.1111/gcb.12724. 25163596

[gcb70834-bib-0076] Tang, R. , B. He , H. W. Chen , et al. 2022. “Increasing Terrestrial Ecosystem Carbon Release in Response to Autumn Cooling and Warming.” Nature Climate Change 12, no. 4: 380–385. 10.1038/s41558-022-01304-w.

[gcb70834-bib-0077] Thornley, J. H. M. 1970. “Respiration, Growth and Maintenance in Plants.” Nature 227, no. 5255: 304–305. 10.1038/227304b0. 5428208

[gcb70834-bib-0078] Vargas, R. , and M. F. Allen . 2008. “Environmental Controls and the Influence of Vegetation Type, Fine Roots and Rhizomorphs on Diel and Seasonal Variation in Soil Respiration.” New Phytologist 179, no. 2: 460–471. 10.1111/j.1469-8137.2008.02481.x. 19086292

[gcb70834-bib-0079] Wang, X. , L. Liu , S. Piao , et al. 2014. “Soil Respiration Under Climate Warming: Differential Response of Heterotrophic and Autotrophic Respiration.” Global Change Biology 20, no. 10: 3229–3237. 10.1111/gcb.12620. 24771521

[gcb70834-bib-0080] Ward, S. E. , R. D. Bardgett , N. P. McNamara , and N. J. Ostle . 2009. “Plant Functional Group Identity Influences Short‐Term Peatland Ecosystem Carbon Flux: Evidence From a Plant Removal Experiment.” Functional Ecology 23, no. 2: 454–462. 10.1111/j.1365-2435.2008.01521.x.

[gcb70834-bib-0081] Ward, S. E. , K. H. Orwin , N. J. Ostle , et al. 2015. “Vegetation Exerts a Greater Control on Litter Decomposition Than Climate Warming in Peatlands.” Ecology 96, no. 1: 113–123. 10.1890/14-0292.1. 26236896

[gcb70834-bib-0082] Wu, J. , N. T. Roulet , J. Sagerfors , and M. B. Nilsson . 2013. “Simulation of Six Years of Carbon Fluxes for a Sedge‐Dominated Oligotrophic Minerogenic Peatland in Northern Sweden Using the McGill Wetland Model (MWM).” Journal of Geophysical Research: Biogeosciences 118, no. 2: 795–807. 10.1002/jgrg.20045.

[gcb70834-bib-0083] Wu, Y. , D. L. Verseghy , and J. R. Melton . 2016. “Integrating Peatlands Into the Coupled Canadian Land Surface Scheme (CLASS) v3.6 and the Canadian Terrestrial Ecosystem Model (CTEM) v2.0.” Geoscientific Model Development 9, no. 8: 2639–2663. 10.5194/gmd-9-2639-2016.

[gcb70834-bib-0084] Yao, H. , H. Peng , B. Hong , et al. 2022. “Environmental Controls on Multi‐Scale Dynamics of Net Carbon Dioxide Exchange From an Alpine Peatland on the Eastern Qinghai‐Tibet Plateau.” Frontiers in Plant Science 12: 791343. 10.3389/fpls.2021.791343. 35069648 PMC8767066

[gcb70834-bib-0085] Yu, Z. 2011. “Holocene Carbon Flux Histories of the World's Peatlands: Global Carbon‐Cycle Implications.” Holocene 21, no. 5: 761–774. 10.1177/0959683610386982.

[gcb70834-bib-0086] Zeh, L. , J. Limpens , B. Erhagen , L. Bragazza , and K. Kalbitz . 2019. “Plant Functional Types and Temperature Control Carbon Input via Roots in Peatland Soils.” Plant and Soil 438, no. 1: 19–38. 10.1007/s11104-019-03958-6.

