# Supplementary material for: The Separate Roles of Vascular Plants and Sphagnum Mosses in Regulating the Net CO2 Exchange in a Boreal Peatland During Key Phenological Phases
Source: Glob Chang Biol. 2026 Apr 3;32(4):e70834. doi: 10.1111/gcb.70834 (PMC13047721; doi:10.1111/gcb.70834)
Supplement: Supplementary file 1 — Figure S1: Pictures of the closed shading umbrella during dark measurements on vegetated plots. Figure S2: Filtering criteria for discarding poor quality fluxes. Figure S3: Treatment‐specific nighttime flux correction. Figure S4: Measured and modelled chamber CO2 fluxes. Table S1: Total number of potential chamber measurements per growing season, amount of data removed during quality‐control filtering (%) and coefficient of determination (R 2) of predicted to gap‐filled fluxes for holdout sets during 10‐fold cross validation using XGBoost per chamber. Table S2: Maximum green vascular plant biomass (g DW m‐2) within the automated natural chamber plots per growing season 2021–2023. Table S3: Results of stepwise general linear model for abiotic (PPFD, photosynthetic photon flux density; T a, air temperature; WTL, water table level) and biotic factors (gcc, green chromatic coordinate used as a proxy for greenness) explaining daily variation of gross primary production (GPP) and autotrophic respiration (RA) of Sphagnum mosses (M) and vascular plants (V). Significance levels are denoted with asteriks * (p‐value < 0.05), ** (< 0.01) and *** (< 0.001). [file GCB-32-e70834-s001.pdf]

Supporting Information (S):

The separate roles of vascular plants and *Sphagnum* mosses in regulating the net CO_2 exchange in a boreal peatland during key phenological phases

Antonia Hartmann[1], Kyohsuke Hikino[1], Lukas Guth[1,2], Gillian Simpson[1], Järvi Järveoja[1], Mats B. Nilsson[1], Matthias Peichl[1]

[1]Department of Forest Ecology and Management, Swedish University of Agricultural Sciences, Sweden

[2]Thünen-Institute of Climate-Smart Agriculture, Bundesallee 65, 38116 Braunschweig, Germany

Supporting Information Section 1: Description of the chamber measurement cycle

One measurement cycle at each vegetated plot consisted of five consecutive steps: (i) 1 min flushing of the sample tubing with ambient air while the chamber was still open; followed by (ii) 3 min of concentration measurements under ambient light during which the chamber was closed; (iii) 2 min of flushing the sampling tubing after the chamber opened again; followed by (iv) 3 min (13 min in 2023 due to a parallel experiment) of repeated concentration measurements under dark conditions during which the chamber and the shading umbrella were closed; and (v) concluded by another 1 min flushing of the sampling tubing during which the chamber and umbrella were open again. The RH chamber was closed for 8 min, of which the first 3 min of concentration measurement were used for the flux calculation. This resulted in a total measurement cycle of 10 min (20 min in 2023) per chamber and thus one completed measurement round every 2 hours (4 hours in 2023) of the 12 chambers. The chamber measurement sequence was kept constant, that is, cycling from group one to group four, and within each group from the natural to the moss and at last to the RH plots. The dark measurement of the vegetated chambers was prolonged in 2023 to accommodate another project that required longer closure times to develop Keeling plots (unpublished data). However, only the first 3 min of concentration measurement were used from these longer closure periods to ensure consistency in the flux data acquisition in this study.

Figure S1: Pictures of the closed shading umbrella during dark measurements on vegetated plots

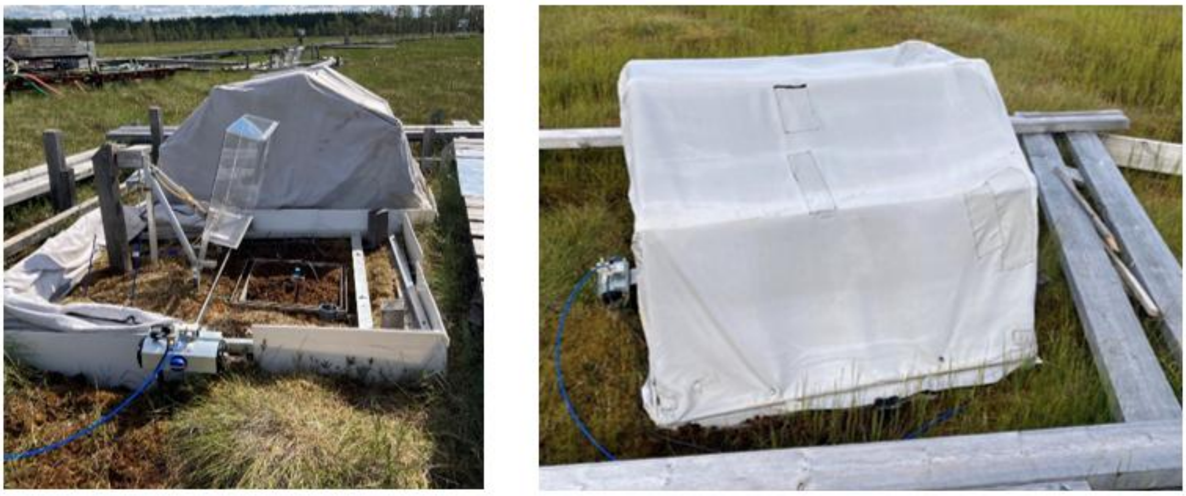

Figure S1: Left: image forefront shows a transparent chamber (open) with open "umbrella" and in the adjacent plot an ongoing dark measurement with closed transparent chamber, covered by a shading umbrella on vegetated plots (i.e., natural and moss plot) with an aluminium base frame (1.2 x 1.6 m). Right: frontal image of the closed shading umbrella.

Figure S2: Filtering criteria for discarding poor quality fluxes

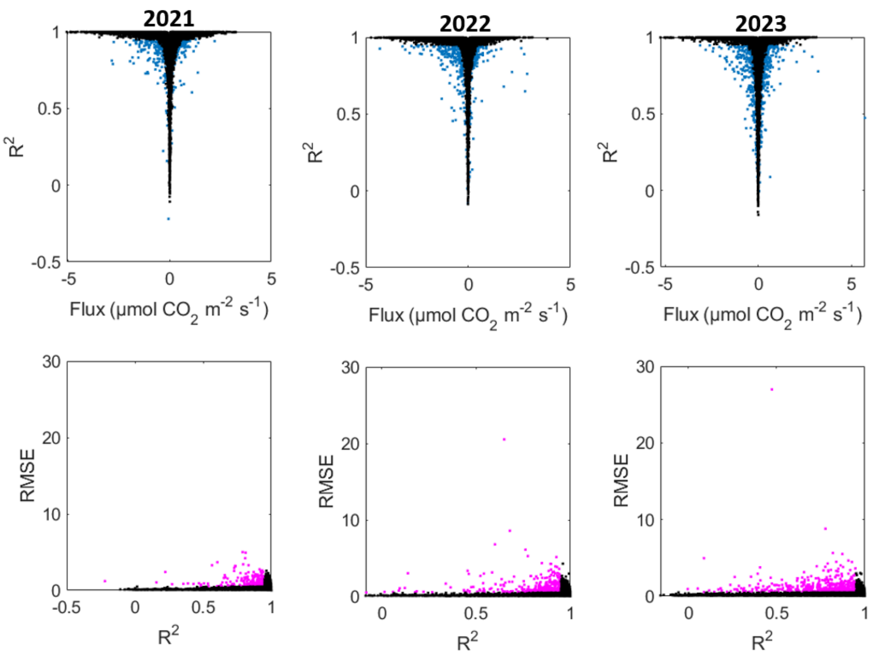

Figure S2: Quality filtering based on coefficient of determination (R^2, upper panel) and root mean square error (RMSE, lower panels) thresholds for 2021-2023. Colored and back symbols indicate poor ($R^2 < 0.95$ and RMSE > 0.5) and good quality fluxes.

Figure S3: Treatment-specific nighttime flux correction

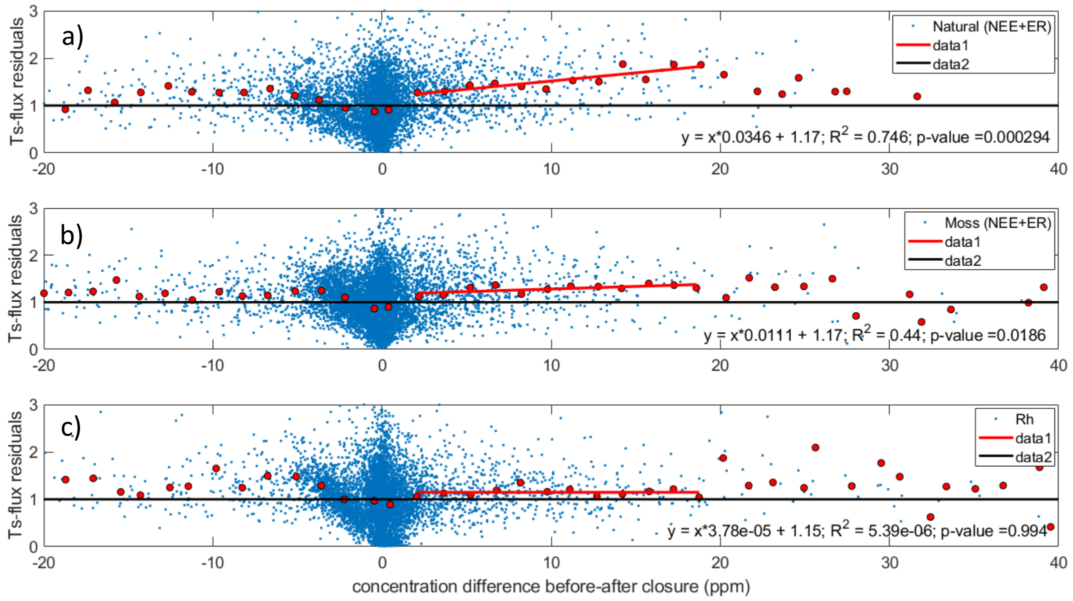

Figure S3: Regression between the concentration difference before and after chamber closure and the residuals from the relationship of nighttime fluxes and soil temperature; Ts at 2cm (blue symbols) for a) natural plots, b) moss plots and c) RH plots. The slope of the linear fit (red line) of the block averages (red markers) was used to correct nighttime fluxes with a before and after chamber closure concentration difference of < 20 ppm.

Figure S4: Measured and modelled chamber CO_2 fluxes

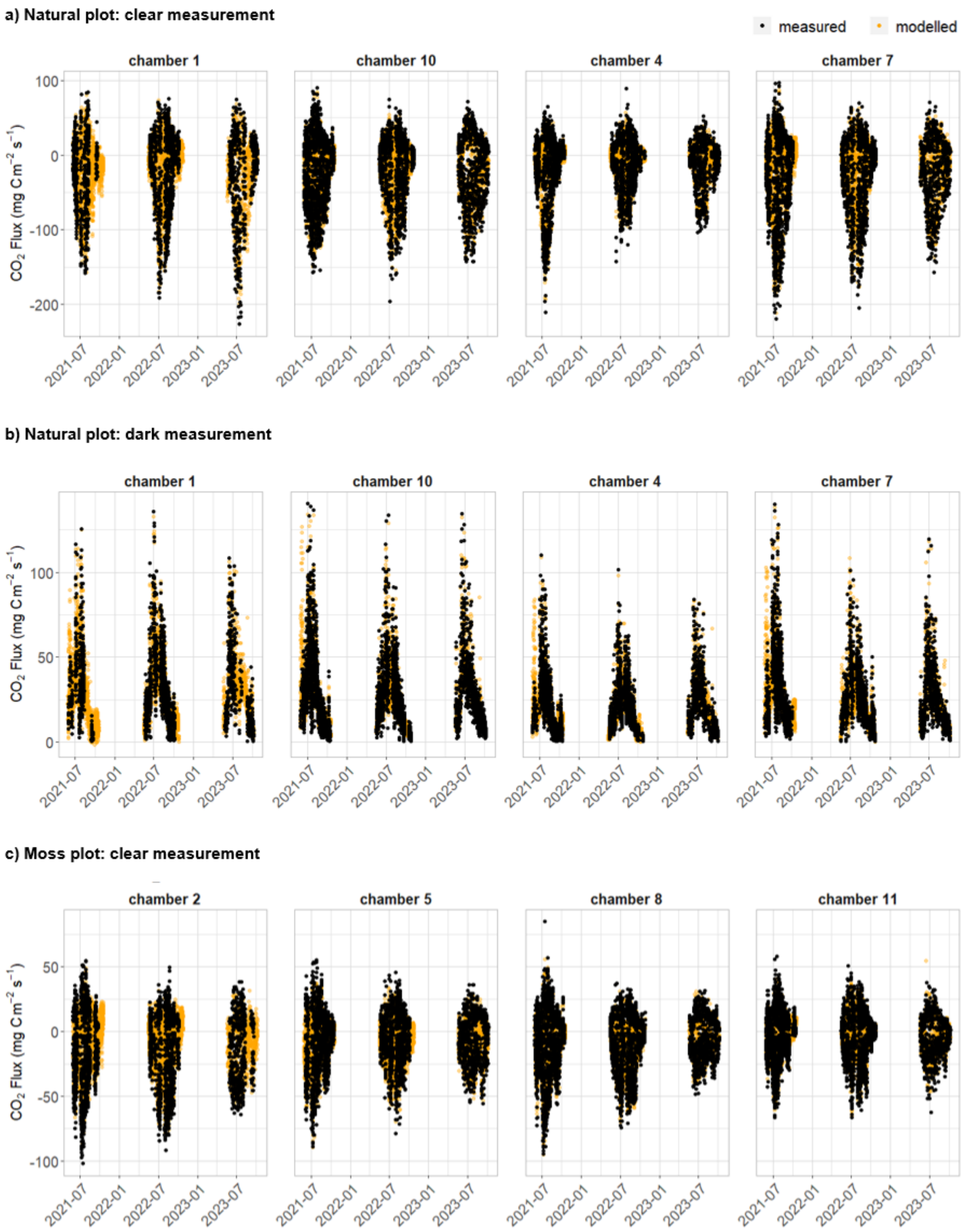

d) Moss plot: dark measurement

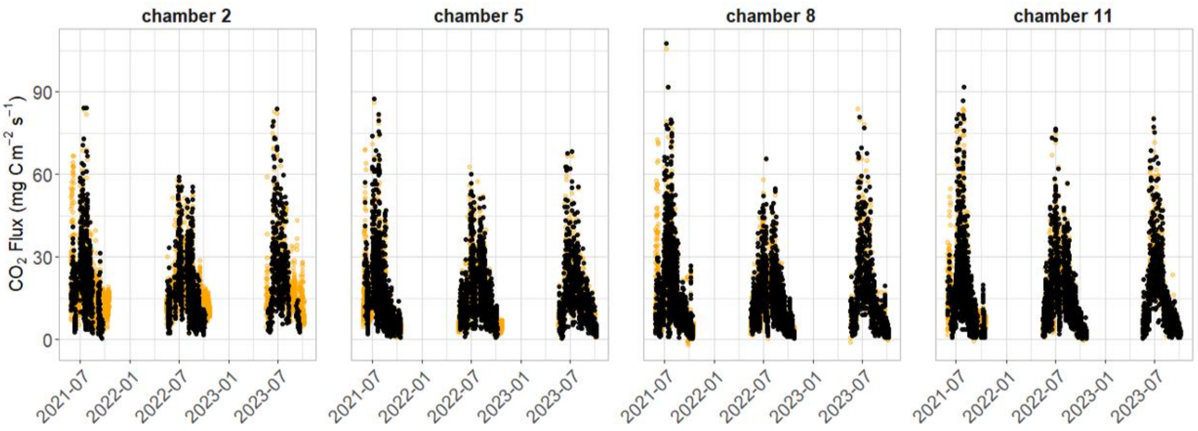

e) RH plot

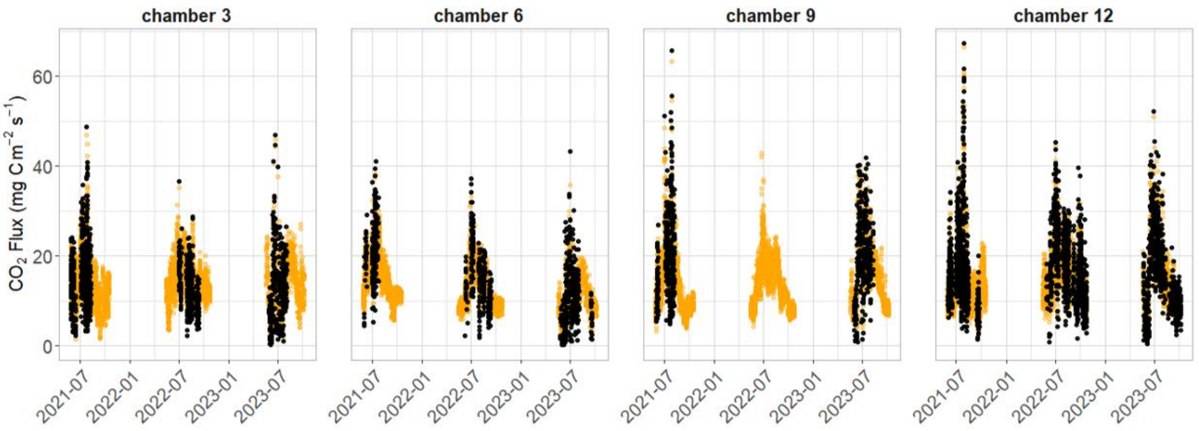

Figure S4: 2-hourly to 4-hourly measured (black) and modelled (orange) fluxes of a) clear measurement and b) dark measurement of natural plots, a) clear measurement and b) dark measurement of vascular plant removel plot (i.e., moss plot) and e) total biomass removal plot (i.e., RH plot).

Table S1: Total number of potential chamber measurements per growing season, amount of data removed during quality-control filtering (%) and coefficient of determination (R^2) of predicted to gap-filled fluxes for holdout sets during 10-fold cross validation using XGBoost per chamber.

		**2021**		**2022**		**2023**		
Treatment	chamber number	Total number of potential chamber measurements	data removed during quality filtering (%)	Total number of potential chamber measurements	data removed during quality filtering (%)	Total number of potential chamber measurements	data removed during quality filtering (%)	R^2
Natural plots, clear measurement	1	1715	69.3	1941	48.5	850	40.1	0.81
	4	1715	23.7	1941	41.7	850	22.4	0.85
	7	1715	34.5	1941	35	850	17.8	0.85
	10	1715	18.4	1941	41.4	850	9.9	0.8
**Total:**		**6860**	**36.5**	**7764**	**41.7**	**3400**	**22.5**	**0.83**
Natural plots, dark measurement	1	1715	79.3	1941	49.8	850	51.1	0.8
	4	1715	36.8	1941	42.6	850	25.7	0.86
	7	1715	44.6	1941	43	850	18.4	0.86
	10	1715	27.5	1941	39.5	850	11.4	0.84
**Total:**		**6860**	**47**	**7764**	**43.7**	**3400**	**26.6**	**0.84**
Moss plot, clear measurement	2	1715	42.9	1941	48.6	850	41.2	0.85
	5	1715	27.3	1941	44.1	850	15.1	0.76
	8	1715	16.7	1941	23.5	850	11.4	0.82
	11	1715	22.2	1941	23.1	850	17.9	0.79
**Total:**		**6860**	**27.3**	**7764**	**34.8**	**3400**	**21.4**	**0.81**
Moss plot, dark measurement	2	1715	58.3	1941	52.5	850	48.5	0.81
	5	1715	46.5	1941	43.3	850	21.1	0.77
	8	1715	38.7	1941	32.6	850	20.2	0.83
	11	1715	41	1941	30.3	850	16.2	0.84
**Total:**		**6860**	**46.1**	**7764**	**39.7**	**3400**	**26.5**	**0.81**

		2021		2022		2023		
Treatment	chamber number	Total number of potential chamber measurements	data removed during quality filtering (%)	Total number of potential chamber measurements	data removed during quality filtering (%)	Total number of potential chamber measurements	data removed during quality filtering (%)	R^2
	3	1715	72.9	1941	77.3	850	62.8	0.63
	6	1715	88.5	1941	82.1	850	61.8	0.63
	9	1715	77.9	1941	100	850	59.5	0.7
RH plot	12	1715	55.5	1941	54.2	850	24.8	0.81
**Total:**		**6860**	**73.7**	**7764**	**78.4**	**3400**	**52.2**	**0.69**

Table S2: Maximum green vascular plant biomass (g DW m^{-2}) within the automated natural chamber plots per growing season 2021-2023.

Chamber	Year	*Eriophorum vaginatum*	*Trichophorum cespitosum*	*Carex pauciflora*	*Scheuchzeria palustris*	*Vaccinium oxycoccos*	*Andromeda polifolia*
1	2021	10.6	0.0	0.0	63.7	6.0	11.3
	2022	12.7	3.5	1.5	70.3	2.8	7.6
	2023	30.1	11.6	0.0	57.2	5.9	4.9
4	2021	17.3	3.3	0.9	24.9	6.6	3.6
	2022	15.9	8.2	0.0	23.4	3.9	8.8
	2023	14.9	8.4	0.0	15.8	8.3	8.7
7	2021	11.2	0.8	0.4	8.3	4.9	34.3
	2022	16.7	1.2	0.0	4.7	5.2	28.8
	2023	87.2	8.2	0.2	10.9	11.3	22.5
10	2021	16.2	0.0	0.7	5.8	10.7	19.8
	2022	28.9	0.0	0.4	11.8	11.2	16.9
	2023	57.4	1.5	1.4	8.2	8.6	14.8

Table S3: Results of stepwise general linear model for abiotic (PPFD, photosynthetic photon flux density; Ta, air temperature; WTL, water table level) and biotic factors (gcc, green chromatic coordinate used as a proxy for greenness) explaining daily variation of gross primary production (GPP) and autotrophic respiration (RA) of *Sphagnum* mosses ($_M$) and vascular plants ($_V$). Significance levels are denoted with asteriks * (p-value < 0.05), ** (<0.01) and *** (< 0.001).

	GPP_M											
	**Growing season**			**Green-up**			**Peak**			**Senescence**		
**Deviance R^2**	**0.12**			**0.22**			**0.25**			**0.13**		
	effect size	standard deviation	signifi-cance level	effect size	standard deviation	signifi-cance level	effect size	standard deviation	signifi-cance level	effect size	standard deviation	signifi-cance level
gcc	-	-	-	-0.26	0.08	**	-	-	-	-	-	-
PPFD	-0.11	0.02	***	-	-	-	-0.16	0.04	***	-0.11	0.04	**
WTL	-	-	-	-	-	-	-	-	-	0.09	0.03	**
Ta	-	-	-	-	-	-	-	-	-	-	-	-
gcc:PPFD	-0.07	0.02	**	-	-	-	-	-	-	-	-	-
gcc:WTL	-0.13	0.03	***	-0.29	0.08	***	0.17	0.03	***	-	-	-
gcc:Ta	-	-	-	-	-	-	-	-	-	-	-	-
PPFD:WTL	-	-	-	-	-	-	-	-	-	-	-	-
WTL:Ta	-	-	-	0.23	0.08	**	-	-	-	-0.15	0.03	***
PPFD:Ta	-	-	-	-	-	-	-0.08	0.03	*	-	-	-
gcc:PPFD:WTL	-	-	-	-	-	-	-	-	-	-	-	-
gcc:PPFD:Ta	-	-	-	-	-	-	-	-	-	-	-	-
gcc:WTL:Ta	-	-	-	-	-	-	-	-	-	-	-	-
PPFD:WTL:Ta	-	-	-	-	-	-	-	-	-	-	-	-

**GPP_V**												
	**Growing season**			**Green-up**			**Peak**			**Senescence**		
**Deviance R2**	**0.36**			**0.37**			**0.36**			**0.21**		
	effect size	standard deviation	signifi-cance level	effect size	standard deviation	signifi-cance level	effect size	standard deviation	signifi-cance level	effect size	standard deviation	signifi-cance level
gcc	-0.13	0.04	***	-0.75	0.13	***	-0.16	0.05	**	-0.14	0.04	***
PPFD	-0.19	0.04	***	-0.36	0.11	**	-0.32	0.07	***	-0.15	0.03	***
WTL	-	-	-	-	-	-	-	-	-	-0.07	0.04	*
Ta	-	-	-	-	-	-	-	-	-	-	-	-
gcc:PPFD	-0.16	0.04	***	-	-	-	-	-	-	-0.10	0.03	**
gcc:WTL	-0.10	0.04	*	-	-	-	-	-	-	-0.12	0.03	***
gcc:Ta	-	-	-	-	-	-	-0.28	0.06	***	-	-	-
PPFD:WTL	-	-	-	-	-	-	-	-	-	-	-	-
WTL:Ta	0.09	0.04	*	-	-	-	-	-	-	-	-	-
PPFD:Ta	-	-	-	-0.28	0.11	*	-0.13	0.05	**	-	-	-
gcc:PPFD:WTL	-	-	-	-	-	-	-	-	-	-	-	-
gcc:PPFD:Ta	-0.08	0.03	**	0.39	0.11	**	-	-	-	-	-	-
gcc:WTL:Ta	-	-	-	-	-	-	-	-	-	-	-	-
PPFD:WTL:Ta	-	-	-	-	-	-	-	-	-	-	-	-
**RA_M**												
	**Growing season**			**Green-up**			**Peak**			**Senescence**		
**Deviance R2**	**0.48**			**0.92**			**0.48**			**ns**		
	effect size	standard deviation	signifi-cance level	effect size	standard deviation	signifi-cance level	effect size	standard deviation	signifi-cance level	effect size	standard deviation	signifi-cance level
gcc	0.19	0.08	*	0.27	0.11	*	-	-	-	-	-	-
PPFD	-	-	-	0.34	0.10	**	-0.16	0.07	*	-	-	-
WTL	-	-	-	-0.81	0.08	***	-0.16	0.07	*	-	-	-
Ta	0.24	0.08	**	0.38	0.06	***	0.26	0.07	**	-	-	-
gcc:PPFD	0.32	0.13	*	0.70	0.11	***	-	-	-	-	-	-
gcc:WTL	0.35	0.08	***	-	-	-	-0.30	0.11	**	-	-	-

gcc:Ta	-	-	-	-	-	-	-	-	-	-	-	-
PPFD:WTL	0.95	0.30	**	-1.04	0.14	***	-	-	-	-	-	-
WTL:Ta	-	-	-	-	-	-	-	-	-	-	-	-
PPFD:Ta	-	-	-	-	-	-	0.35	0.07	***	-	-	-
gcc:PPFD:WTL	0.84	0.20	***	-	-	-	-	-	-	-	-	-
gcc:PPFD:Ta	-	-	-	-	-	-	-	-	-	-	-	-
gcc:WTL:Ta	-	-	-	-	-	-	-	-	-	-	-	-
PPFD:WTL:Ta	-0.39	0.12	**	-	-	-	-	-	-	-	-	-
**RA_V**												
	**Growing season**			**Green-up**			**Peak**			**Senescence**		
**Deviance R2**	**0.62**			**0.83**			**0.55**			**0.57**		
	effect size	standard deviation	signifi-cance level	effect size	standard deviation	signifi-cance level	effect size	standard deviation	signifi-cance level	effect size	standard deviation	signifi-cance level
gcc	0.21	0.03	***	0.22	0.04	***	0.15	0.03	***	0.42	0.08	***
PPFD	-	-	-	-	-	-	-0.07	0.03	**	-0.18	0.09	*
WTL	-0.17	0.03	***	-0.25	0.06	***	-	-	-	-	-	-
Ta	0.35	0.03	***	0.20	0.05	***	0.16	0.03	***	0.31	0.07	***
gcc:PPFD	-	-	-	-	-	-	-	-	-	-	-	-
gcc:WTL	-	-	-	0.14	0.05	*	-	-	-	-	-	-
gcc:Ta	-	-	-	-	-	-	0.12	0.03	***	-	-	-
PPFD:WTL	-	-	-	-	-	-	-	-	-	-	-	-
WTL:Ta	-	-	-	-	-	-	-	-	-	-	-	-
PPFD:Ta	-	-	-	-	-	-	-	-	-	-	-	-
gcc:PPFD:WTL	-	-	-	-0.10	0.05	*	-	-	-	-0.20	0.09	*
gcc:PPFD:Ta	-	-	-	-	-	-	-	-	-	-	-	-
gcc:WTL:Ta	0.12	0.03	***	-	-	-	-	-	-	0.14	0.07	*
PPFD:WTL:Ta	-	-	-	-	-	-	-	-	-	-	-	-
